# Rare, functional, somatic variants in gene families linked to cancer genes: GPCR signaling as a paradigm

**DOI:** 10.1038/s41388-019-0895-2

**Published:** 2019-07-23

**Authors:** Francesco Raimondi, Asuka Inoue, Francois M. N. Kadji, Ni Shuai, Juan-Carlos Gonzalez, Gurdeep Singh, Alicia Alonso de la Vega, Rocio Sotillo, Bernd Fischer, Junken Aoki, J. Silvio Gutkind, Robert B. Russell

**Affiliations:** 1 BioQuant, Heidelberg University, Im Neuenheimer Feld 267, 69120 Heidelberg, Germany; 2 Heidelberg University Biochemistry Centre (BZH), Im Neuenheimer Feld 328, 69120 Heidelberg, Germany; 3 Graduate School of Pharmaceutical Science, Tohoku University, Sendai, 980-8578 Miyagi Japan; 4 Advanced Research & Development Programs for Medical Innovation (PRIME), Japan Agency for Medical Research and Development (AMED), Chiyoda-ku, Tokyo, 100-0004 Japan; 5 Computational Genome Biology, German Cancer Research Center (DKFZ), 69120 Heidelberg, Germany; 6 Division of Molecular Thoracic Oncology, German Cancer Research Center (DKFZ), Translational Lung Research Center (TLRC), Member of the German Center for Lung Research (DZL), 69120 Heidelberg, Germany; 7 Moores Cancer Center, University of San Diego, San Diego, La Jolla, CA 92093 USA

**Keywords:** Cancer genomics, Cancer genomics, Cell signalling, Cell signalling

## Abstract

Oncodriver genes are usually identified when mutations recur in multiple tumours. Different drivers often converge in the activation or repression of key cancer-relevant pathways. However, as many pathways contain multiple members of the same gene family, individual mutations might be overlooked, as each family member would necessarily have a lower mutation frequency and thus not identified as significant in any *one-gene-at-a-time* analysis. Here, we looked for mutated, functional sequence positions in gene families that were mutually exclusive (in patients) with another gene in the same pathway, which identified both known and new candidate oncodrivers. For instance, many inactivating mutations in multiple G-protein (particularly G_i/o_) coupled receptors, are mutually exclusive with Gα_s_ oncogenic activating mutations, both of which ultimately enhance cAMP signalling. By integrating transcriptomics and interaction data, we show that the G_s_ pathway is upregulated in multiple cancer types, even those lacking known *GNAS* activating mutations. This suggests that cancer cells may develop alternative strategies to activate adenylate cyclase signalling in multiple cancer types. Our study provides a mechanistic interpretation for several rare somatic mutations in multi-gene oncodrivers, and offers possible explanations for known and potential off-label cancer treatments, suggesting new therapeutic opportunities.

## Introduction

Cancer genome sequencing projects have revealed a growing list of genes and mutations driving tumor initiation and progression [1–5]. However, even when an oncodriver role is well established, it remains difficult to discriminate driver from passenger mutations, especially when they are rare [6]. This task is more daunting when sparse somatic mutations affect genes not previously linked to cancer by standard approaches based on positive selection. A recent systematic survey of somatic mutations under positive selection estimated that nearly half of driving events are found in genes not yet linked to cancer [7]. With genome sequencing entering clinical practice, more powerful approaches able to identify rare driver mutations are therefore required.

Analysis of mutated genes, in the context of pathways and networks [8] or three-dimensional structures [5, 9], has aided the detection of driver variants, also illuminating mechanisms adopted by cancer cells for tumor growth and spread. For example, frequently mutated oncodrivers rarely participate to the same interaction interface [10]: e.g., *TP53* is mutated in many cancers, but its most common interaction partners are not. However, there are instances where mutations in different parts of one pathway appear to be responsible for a common cancer. For example, mutations affecting *KRAS*/*NRAS* and *BRAF* are often found mutually exclusively along the MAP kinase cascade pathway, particularly in melanoma, colorectal, lung and pancreatic cancers [11]. Analysis of cancer genomes has revealed many examples of mutually exclusive events concurring with specific cancer phenotypes [12].

Given the redundancy of many biological processes, where multiple proteins can modulate a common up- or downstream partner, it is possible that multiple genes could essentially replace mutations in a single common cancer gene. Indeed, several known oncodrivers have multiple regulators. For example, the phosphoinositide 3-kinase (PI3K) pathway, shows great redundancy in that downstream target PI3Ks can be activated by many upstream signals transduced by tyrosine kinases and G-protein coupled receptors [13].

**Fig. 1:**
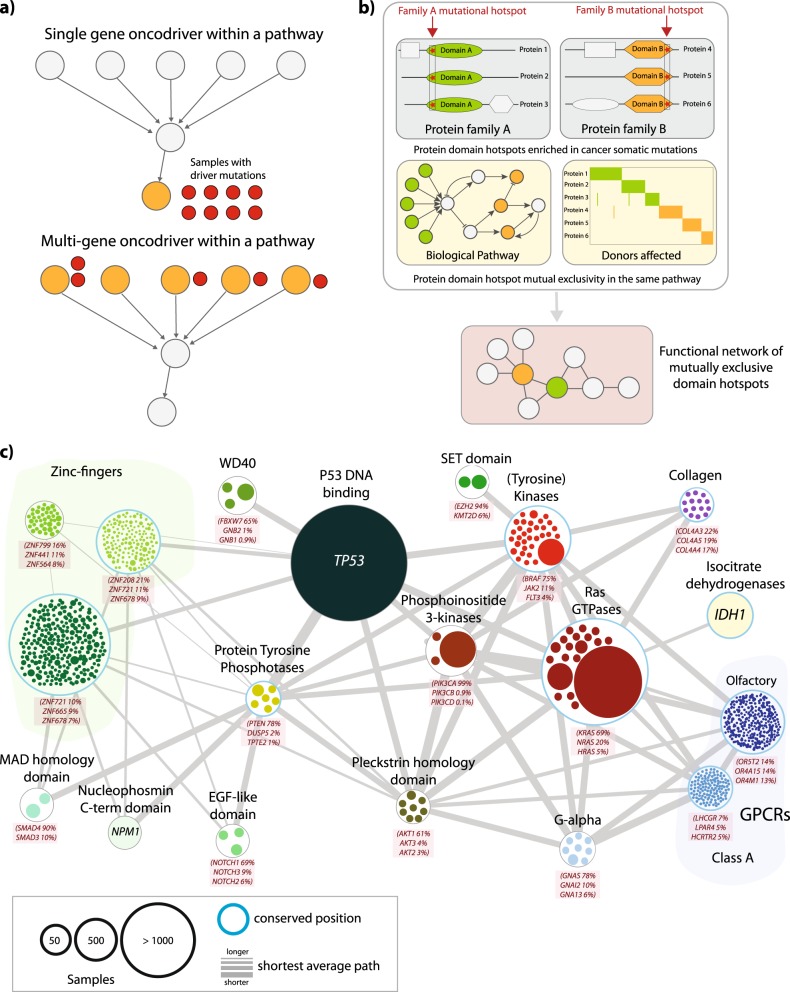
**a** Multi-gene oncodriver hypothesis. **b** Analysis workflow. **c** Network showing functionally related protein families with members in common Reactome pathways (nodes) displaying significantly enriched, mutually exclusive mutated positions (edges) pan-cancer. Node diameter is proportional to the total number of nonsynonymous mutations (number of unique samples) for a family member; thicker cyan borders indicate families where at least one highly conserved position is significantly mutated. Inside each node, mutated members of a given family are displayed, with a diameter proportional to the number of mutations. Edge thickness is proportional to average shortest paths between the two families

Instances of this *multi-gene oncodriver* phenomenon would often be overlooked as mutations in multiple upstream genes necessarily have lower frequencies, and thus would not be identified as statistically significant in any *one-gene-at-a-time* analysis. Here we searched for such examples, by grouping all genes into protein domain families and looking for pairs of functionally linked families showing exclusivity of mutations in particular cancers (Fig. 1a). We identify several instances of this phenomenon, involving both oncogenes and tumor suppressors, including sparsely mutated, genes not previously linked to cancer.

## Results

### A network of mutually exclusive, variant-enriched protein family positions in cancer

We defined a set of interacting gene families as those sharing a common protein domain [14], and residing in the same pathway (from Reactome; [15]). In each pair of families, we then sought protein alignment positions that were mutually exclusive with regard to somatic, non-synonymous cancer variants (Methods; Fig. 1b), either in specific cancers or across all of them (pan-cancer). We considered 1.9 M simple somatic non-synonymous mutations, from 414 different cancer subtypes (from 37 primary tissues and 49 histologies). A total of 794k mutations (41% of the total) affect 282k domain positions from 5.7k human protein families (92% of the total). We defined significantly enriched domain positions according to a gene-level background model (to correct for genes with different mutational burden; Fig. S1 and Tables S1, S2), and retained pairs of positions displaying significant enrichment in a pathway-level background model (Tables S3, S4; see Methods). We chose this model, similar to one used in another recent study [16], and not models designed to detect positive selection in individual genes [3], as we were seeking positions across families: using other models would have filtered out many rare variants that were precisely what we sought. Changing the parameters of the analysis (i.e., mutation thresholds, set of pathways considered, background model) led only to moderate changes in the number of identified positions. The number of dubious genes (i.e., those more tolerant to mutations [17]) uncovered also does not vary using different parameters and it is moreover always much lower than when using the 1000 Genomes set (see Methods and Table S5).

It is possible that the mutually exclusive pairs we detect arise because mutations in both partner proteins in one sample would simply not be tolerated in living cells. While we cannot rule this out in all instances, evidence that this is not the case comes from the fact that we see co-occurrence of mutations, in at least one sample, for 56% of all hotspot pairs (i.e., in addition to the observed exclusivity). This suggests that the mutations are at least, in principle, tolerated together. Moreover, few of the pairs we identified involve interactions between well-established oncodrivers, but most often involve one oncodriver and a larger family of proteins.

Our pan-cancer analysis revealed 414 significantly mutated (Binomial *q* < 0.1) and mutually exclusive (Fischer’s exact *q* < 0.1) pairs, involving a total of 86 positions from 23 families in 55 pathways (Fig. 1c), affecting 9.1k (43% of the total) samples. The three most represented pathways were tyrosine kinase signalling, generic transcription, and GPCR downstream signalling (Fig. S2; Table S3). We found that domains with significant mutated positions have similar fractions of highly conserved positions compared to those with no mutations (*p* = 0.17; Table S6). Moreover, only a minority (22%) of the top 50 largest sequence families in the human proteome have at least one significantly mutated positions, ruling out a bias owing to family size (see Table S6).

The process of identifying variant-enriched, mutually exclusive positions also highlights functional positions, as they are enriched for sites that bind to other molecules compared to random selections [18] (Methods). Nearly half of mutually exclusive position pairs (171 out of 336 matched to an interaction interface) have similar predicted functional consequences in the sense of targeting protein, small-molecule or nucleic acid binding sites (Fig. S3). 9% (of 5188) gene-pairs share a specific protein and 39% share a small-molecule interaction partner, indicating a more precise functional overlap (Table S7, see below).

The 1055 genes linked in the network (Fig. 1c), involve several instances of small (even single member) families, which include relations between well-known cancer genes. Not every known oncodriver is found, which is expected since the method is very restrictive: requiring gene family pairs each to have a specific position enriched in variants in a mutually exclusive fashion within at least one common pathway. Nevertheless, some well-known examples are in this network. For example, *KRAS* p.G12D with *BRAF* p.V600E, and *AKT1* p.E17K with *PIK3CA* p.E545K. The approach also revealed candidate multi-gene oncodrivers, namely where positional variants scattered across a large family show mutual exclusivity with positions in a single protein downstream.

### Mutations in multiple transcription factors are mutually exclusive with *TP53*, representing potential tumor suppressors

A major part of the network (Fig. 1c) relates to tumor suppressor activities mediated by transcription factors, particularly *TP53*. Mutations in several genes with transcriptional regulatory functions (i.e., the left side of Fig. 1c) are mutually exclusive (e.g., zf-C2H2 or zf-C2H6 with either P53 or EGF domains). Several known oncodrivers involved in this functional subnetwork are genes with known tumor suppressor properties, such as *TP53*, *PTEN*, *FBXW7, SMAD3* and *SMAD4*.

**Fig. 2:**
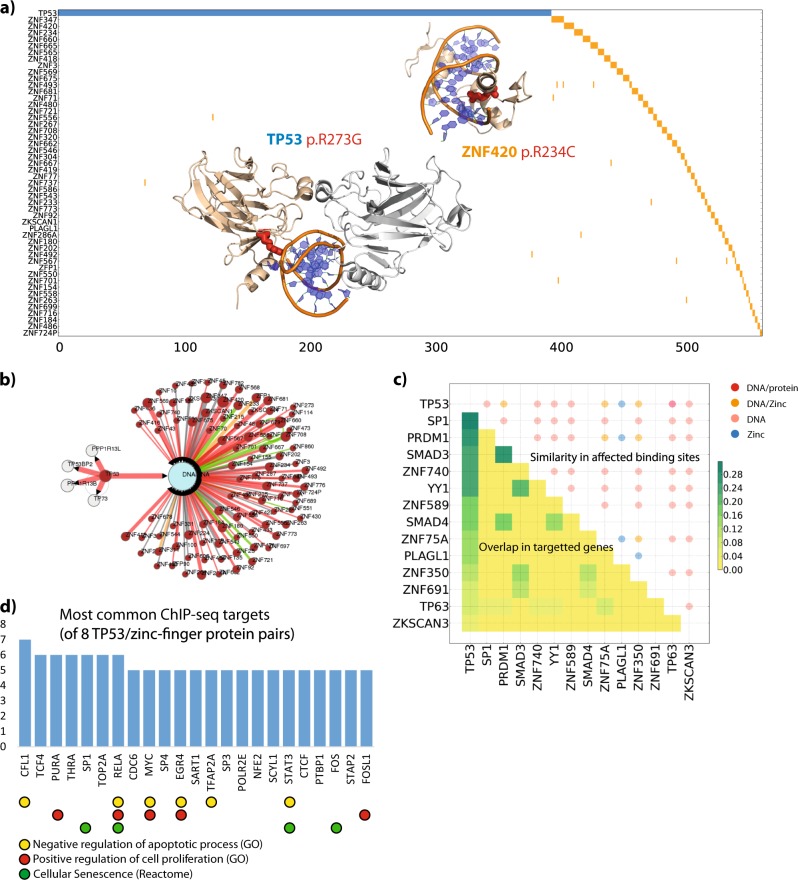
TP53/Zinc-finger mutually exclusive mutations: **a** 2D representation of patients (columns) affected by TP53 p.R273 mutations (blue) and zf-C2H2 r12 position (orange). Genes (rows) are sorted based on mutation frequency, showing the top 50 most mutated zinc fingers. 3D cartoon representations of *TP53* (PDB ID: 2AC0) and *ZNF420* (PDB ID: 1MEY) highlighting the mutated arginines as red sticks; Note that the structures are placed so they do not cover any non-white portion of the plot (i.e., in blank parts of the plot). **b** Mechismo network representation of predictions for the mutations in TP53 and zinc-fingers. Mutated genes are magenta; DNA is cyan. Red and green links indicate interaction disabling and enabling predicted effects. **c** Correlation between transcription factor mutations predicted effect and similarity of target genes. The upper half of the matrix shows the proportion of target genes (Jaccard score) shared by two transcription factors. Only the top 13 genes more similar to *TP53* are shown, along with their Jaccard scores (considering all the possible pairs). Circles in the upper half of the matrix indicate overlap of predicted mutation (Mechismo) effects. Colors indicate the class of interactor affected and size is proportional to the number of mutually exclusive positions with the same predicted outcome. **d** Counts of the most common target genes shared across 8 TP53/zinc-finger pairs with Reactome and gene-ontology (GO) groups indicated below

In addition, many of these relationships involve transcription factors with broadly similar functions, including zinc-fingers with positions that were found to be exclusive with *TP53* counterparts. The majority of positions are residues involved in DNA-binding in both families, where the predicted effect [18] is a loss of binding, suggesting that one could indeed replace the other functionally (Fig. 2a, b). In support of this, several zinc-finger proteins show similarities in transcriptional targets with *TP53*, as identified in ChIP-seq/ChIP-chip data (Methods; Fig. 2c). These include growth promoting genes such as *MYC*, *RELA*, and *STAT3* (Fig. 2d). Remarkably, zinc-fingers *SP1*, *PRDM1*, *ZNF740* and *YY1* all show overlaps of more than 20% with *TP53* target genes (Table S8). Several have already been linked to cancer; for example *SP1* is a prognostic factor for lower survival in gastric cancer [19], *PRDM1* to be a tumor suppressor in lymphomas [20] and *YY1* as an initiator of tumorigenesis in several malignancies [21]. Most of these relationships were statistically significant when considering the large pan-cancer dataset, though several are also seen in specific cancer tissues (Table S9), including, skin and large intestine.

### Multiple GPCRs mutations are exclusive with G-proteins and other oncogenes

The other main component of the pan-cancer network (Fig. 1c, right) relates broadly to signal transduction and contains several known oncogenes (e.g., Ras-family members, tyrosine and serine-threonine kinases, and G proteins). This also involves several hundred GPCR genes that show positional exclusivity with G-protein hotspots. A major contributor to this is the DRY Arginine (R^3.50^, superscript denotes the Ballesteros/Weinstein numbering [22], see Methods), which is the most significantly enriched (*q* = 2 × 10^−35^; Table S1) position, and which shows mutual exclusivity with recurrent mutations of the heterotrimeric Gα family catalytic switch I (SWI) arginine (mainly accounted by GNAS p.R201^G.hfs2.2^, superscript denotes the Common Gα numbering [23]; Fisher exact *q* < 0.001; Table S3).

DRY is an important motif for Class A GPCR activation [24, 25], mediating intra-molecular polar contacts holding receptors inactive until ligand binding [25]. The arginine is recurrently mutated (Table S10), with a total of 94 class A GPCRs having at least one somatic mutation at this arginine in 153 unique samples for a total of 189 non-synonymous mutations (see Table S1). Among them, the most frequently mutated are *HCRTR2*, *GPR174*, *P2RY12* and *LPAR4*. The majority of mutations at the G-alpha SWI Arginine position (Table S11) are found in *GNAS* (109 samples) with others found in *GNAI2* (12), *GNA13* (7), *GNA15* (4) and *GNAQ/11* (6). DRY arginine also displays mutual exclusivity with positions in genes involved in GPCR-mediated downstream signalling including many oncogenes: *AKT1(2,3)* p.E17K, *PIK3CA* p.E545K and p.E542K, *RAC1* p. P29S, *JAK2* p.V617F. (Fig. 1c and Table S3).

**Fig. 3:**
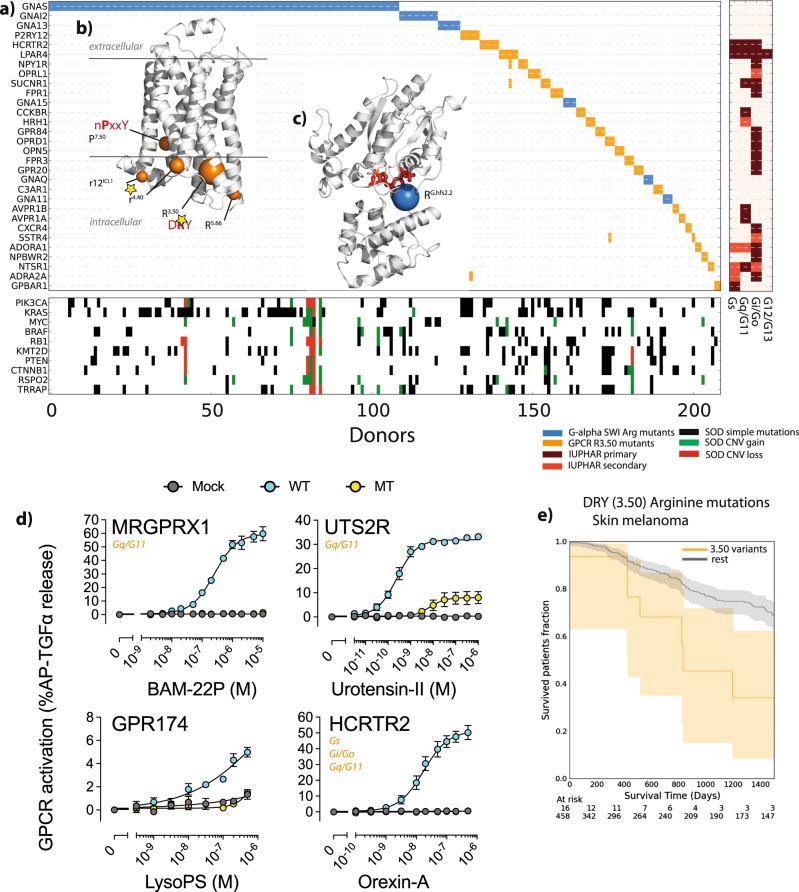
Class A GPCR and G-protein mutually exclusive mutations: **a** as for Fig. 2a, but for GPCR and Gα mutations at either GPCR (DRY) R3.50 or Gα SWI arginine. Only the top 30 mutated genes are shown. **b** GPCR (PDB: 3NYA) and **c** Gα (1AZT) significant positions indicated as spheres centred on Cα atoms and whose diameter is proportional to the number of mutations. The right panel shows GPCRs coupling preferences from IUPHAR (maroon and red indicate primary and secondary coupling respectively). The lower panel shows co-occurring mutations for the top 10 most mutated signalling oncodrivers; **d** Loss of G-protein signalling activity in the DRY mutant GPCRs. HEK293 cells transfected with the alkaline phosphatase-tagged transforming growth factor-α (AP-TGFα)-encoding plasmid together with an empty plasmid (Mock), WT GPCR-encoding plasmid (WT) or DRY-mutant GPCR-encoding plasmid (MT) treated with titrated ligands for 1 h while quantifiying AP-TGFα release into conditioned media. Symbols and error bars represent mean and SEM, respectively, of three to five independent experiments with each measured in triplicates. For MRGPRX1 and HCRTR2, symbols of MT overlap with Mock. Parameters from the concentration-response curves (EC_50_ and *Emax*) are listed in Table S15; **e** Kaplan-Meier curve showing survival analysis for patients affected by R^3.50^ mutations (orange curves) in skin melanoma

Several other GPCR positions also show exclusivity, including P^7.50^ from another highly conserved NPxxY motif, which shows exclusivity with the DRY arginine itself (i.e., intramolecular; Table S3); these motifs cooperate in receptor activation [25]. This proline, together with other positions on the cytosolic side of the GPCR structure (Fig. 3b), also shows mutual exclusivity with the G-alpha SWI arginine and positions from many other downstream oncogenes (Table S3). Moreover, we also found a tendency for mutual exclusivity of GPCR DRY Arginine and G-alpha SWI Arginine in specific tissues, including pancreas (*q* = 0.02; Table S4), large intestine (*p* = 0.02, *q* = 0.23), stomach (*p* = 0.13, *q* = 1) and skin (*p* = 0.25, *q* = 1) (Fig. S5).

The potential importance of these mutations is underscored by their association with poorer survival, either in specific cancers (DRY Arginine in skin melanoma; LogRank *p* = 0.03; Cox’s HR = 1.16; Fig. 3e), or R^4.40^ mutations in all cancers (LogRank *p* = 0.01; Cox’s HR = 1.03; Fig. S4; Tables S12–S14).

The GPCR mutations we find in these cancer samples differ greatly from naturally occurring variants in healthy individuals (Wilcoxon *p* = 0.001; see Fig. S6), which are most often found in Olfactory receptors [26]. Nevertheless, despite the stringent background models adopted, we do see mutual exclusivities involving olfactory receptors (Fig. 1c). Among them is *OR51E2* (Table S3), which is ectopically expressed in melanocytes [27] as well as in primary melanoma and melanoma metastasis [28] and whose activation has been shown to elicit an onco-suppressive effect in prostate carcinoma [29]. Thus, the emerging information suggests that at least a subset of these mutations might have consequences in cancers.

We also found exclusivity between G-alpha positions and downstream signalling partners. In pancreatic tumors, for example, *GNAS* SWI arginine and p.V633M mutations in the downstream adenylate cyclase *ADCY8* are mutually exclusive (Fig. S7 and Table S4) in addition to those affecting DRY Arginine of upstream GPCRs (Fig. S7 and Table S4). In adrenal gland adenomas *GNAS* SWI arginine and p.L207R mutations in the cAMP activated enzyme *PRKACA* are mutually exclusive (Table S4). Remarkably, samples with these *GNAS* or *PRKACA* mutations do not have any other known oncogene or signalling oncodriver mutated (Table S16).

### Widespread dysregulation of GPCR-mediated signaling in cancer

G-alpha SWI arginine mutations are usually oncogenic, leading to increased signaling [30]. The mutual exclusivity would be tantalizingly explained by DRY mutations (or other mutually exclusive GPCR positions) also being activating. This is also an attractive idea as it is known that some mutations, particularly of the DRY motif (e.g., in *ADRA1A* [31] and *AVPR2* [32]), can lead to constitutive activation. However, we found that these positions almost always lead to a loss of function (Fig. 3d), as it is known to be most often the case for mutations to this arginine [33]. Indeed, by experimentally testing the effects of observed DRY arginine mutations in seven representative receptors (selected according to the data in Fig. S5) and the previously described AVPR2 mutations by a TGFα shedding assay [34], we find that these positions almost always lead to a loss of ligand-induced function (Fig. 3d, Fig. S8 and Table S17). Moreover, while AVPR2 mutations, as expected [32], led to constitutive activity, none of the representative receptors with cancer mutations displayed constitutive activation as reported by cAMP assays (Fig. S9).

**Fig. 4:**
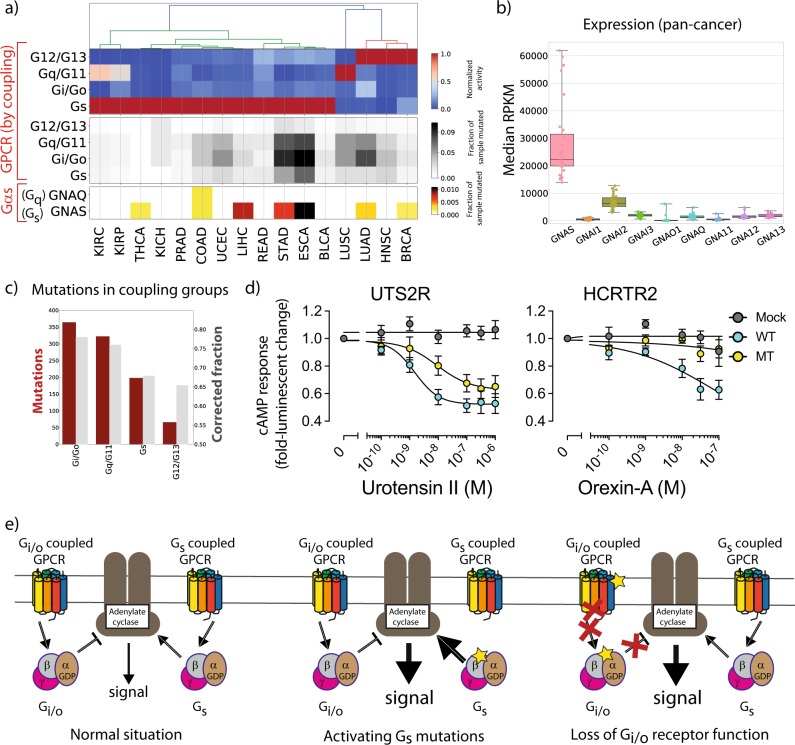
Functional consequence of GPCR mutations: **a** integrative analysis of G-protein activities, hotspot mutations and GPCR deleterious mutations in different TCGA cancer types. Top panel: G-protein activities estimated by combining G-proteins and receptors differential expression levels through coupling information; middle panel: fraction of samples with deleterious GPCR mutations (i.e., either highly conserved residues, stop gains or frameshifts); lower panel: known activating *GNAS* (p.R201) and *GNAQ* (p.Q209) mutations. **b** Median of RPKM values of Gα subunits in 32 TCGA cancer types. **c** Number of unique samples (crimson) and fraction of the coupling group (grey) displaying deleterious mutations pan-cancer; **d** Loss of G_i_ activity in the DRY mutant GPCRs. HEK293 cells transfected with the cAMP biosensor-encoding plasmid and plasmid together with an empty plasmid (Mock), WT GPCR-encoding plasmid (WT) or DRY-mutant GPCR-encoding plasmid (MT) were loaded with D-luciferin for 2 h and treated with titrated ligands in the presence of 10 µM forskolin for 10 min. Luminescent signals were measured before and after ligand addition and data expressed as a change in luminescent counts. Symbols and error bars represent mean and SEM, respectively, of six to seven independent experiments with each measured in duplicates; **e** cartoons of the Adenylate Cyclase pathway regulation summarising the mechanisms described in the text

This seemingly counterintuitive result can be explained by the overall context of GPCR/G-protein mediated signalling. We combined mutation, gene expression and GPCR/G-protein coupling data (i.e., which G-protein is coupled to each GPCR) [35] to estimate the overall activities of the four main G-protein classes in each cancer (Fig. 4a; Methods). This revealed that many cancers, beyond those showing *GNAS* hotspot mutations, display a tendency for widespread up-regulation of G_s_ activity over the other G-proteins, which is particularly evident for G_i/o_ which never prevails in any investigated cancer type (Fig. 4a, top). Indeed, 71% of TCGA cancer types characterized by GNAS activating mutations also show overall higher activity of the G_s_ pathway, which is the most activated in 75% of the considered cancer types (Fig. 4a). Notably, these include all malignancies of the gastro-intestinal tract (i.e., esophagus, stomach, liver, colon, rectum) present in the differential expression panel. More specifically, while G_s_ activity is mainly accounted by higher *GNAS* levels (Fig. 4b), lower G_i/o_ levels are seemingly caused by a diminished expression of either G_i/o_-proteins or their coupled receptors (Figs. S10, S11), which are more frequently affected by deleterious mutations (i.e., stop gains, frameshifts or non-synonymous mutations at highly conserved domain positions; see Methods) in multiple cancers (Fig. 4a; grey in middle panel). Overall, Gi/o coupled receptors are the class hit by the greatest number of deleterious mutations also when considering all cancers (Fig. 4c).

This immediately suggests an explanation for our observation, as deactivating G_i/o_ signalling and activating G_s_ signalling would both lead to an increase in adenylate cyclase activity and cAMP mediated signalling (Fig. 4e). Indeed, we found that cAMP assays for a selection of representative Gi/o coupling GPCRs (UTS2R and HCRTR2) showed that the DRY mutants poorly induced G_i_ activation as measured by loss of inhibitory effect of ligand-induced cAMP luminescent signals (Fig. 4d, Table S18). The much greater expression of *GNAS* relative to the other G protein genes in all cancers (Fig. 4b), would also imply that Gs signalling is prevailing (i.e., even in the absence of any *GNAS* activation) and hence needs to be strictly controlled.

To understand more in details the functional consequences of DRY arginine mutations, we performed differential expression analysis of R^3.50^ mutations in cancer types where it was found at highest recurrence (i.e., skin, gastric and uterine; see Methods). In melanoma 31% (146 out 462) of dysregulated genes in R^3.50^ mutated samples overlap with those of G-protein activating mutations (*GNAQ* p.Q209^G.s3h2.3^ and *GNAI2* p.R179^G.hfs2.2^), indicating at least partial functional equivalence between these mutually exclusive mutation sets and moreover pointing to biological processes characteristic of more aggressive melanomas (e.g., thickening, loss of inhibitory endopeptidase activity [36], see Fig. S12a). Comparative differential expression analysis of R^3.50^ mutations in multiple cancers (i.e., skin, gastric and uterine) revealed a more context specific downstream effect of these mutations, with only 15 genes similarly dysregulated (Fig. S12b). Among these, we found *GNAS-AS1*, the antisense RNA for the *GNAS* locus, which has been reported to regulate its imprinting status [37], thus suggesting an additional layer of regulation of *GNAS*’s activity. Intriguingly, *GNAS-AS1* downregulation by methylation has been associated to colorectal tumorigenesis [38] as well as to lung cancer susceptibility [39].

### An integrated map for GPCR drugs (off-)label prescription

Activation of cAMP signalling via means other than by activating *GNAS* mutations suggests both an explanation for off-label drugs with potential actions against cancer, and the means to identify additional cancer targeting drugs (Table 1, Table S19; Fig. S13). For instance propranolol, an antagonist of the *GNAS* coupled β2 andrenergic receptor (*ADRAB2*), improves prognosis in thick skin melanoma [40] and abrogates EGFR inhibitor resistance acquisition in lung cancer [41]. This suggests that other such antagonists could also prove useful, for example of several other adrenergic receptors (Table 1). In contrast, *agonists* of G_i_ signalling could, in principle, produce similar effects, and interestingly, two drugs targeting G_i_-receptors (Pasireotide targeting somatostatin receptors and adenosine analogs targeting Adenosine A3 receptor) are already in use based on antitumor effects (Table 1). These potential treatments are likely only relevant when cAMP signalling either drives the cancer or where it is linked to therapy resistance, as in melanomas [42], and where there is not overexpression of the GPCR of interest (which could indicate a different mechanism; e.g., *DRD2* in gastric cancers [43]). To aid the search for new therapeutic candidates, we integrated data on GPCR coupling and known antagonists/agonists for all coupling groups (Table 1, Fig. S13).

**Table 1:** G_i/o_-coupled receptor agonists and G_s_-coupled receptor antagonists with known or putative anti-cancer activity

GPCR	Cancers	Agonists	Evidence for therapeutic benefit in cancer	Indications
G_i/o_-coupled receptors with high expression, with no change in expression in tumor compared to wild-type, and with approved agonists				
ADORA3	*BLCA*	2	Antiproliferative effects of adenosine or synthetic agonist in melanoma, prostate, colon and liver carcinomas or lymphoma [70, 71]	Coronary vasodilators, pharmacological stress testing
	HNSC			
	*LUSC*			
	*PRAD*			
	*STAD*			
HTR1D	*STAD*	20	Serotonin analogues are inhibitors of breast cancer cell growth [72]	Agonists used for migrane treatment
S1PR1	*ESCA*	1	Fingolimod efficacy in in-vitro and in-vivo cancer models by inhibition of sphingosine kinase 1 [73] +	Immuno-modulating in Multiple Sclerosis
	*READ*			
	*STAD*			
SSTR1	*ESCA*	1	Pasireotide (somatostatin analogue) can inhibit non-functioning pituitary adenomas and neuroendocrine tumors [74, 75]	Treatment of Cushing’s disease
	KIRP			
	*LIHC*			
	*STAD*			
	*UCEC*			
Gs-coupled receptors with no significant under expression and with approved antagonists				
ADRA2A	*ESCA*	11	None found	Many indications for antagonists, including Parkinson’s, schizophrenia, psychosis, depression and erectile dysfunction
	HNSC			
	*LUSC*			
	*READ*			
	*STAD*			
	*THCA*			
	*UCEC*			
ADRA2B	KICH	13	None found	As above
	KIRC			
	*UCEC*			
ADRA2C	*BLCA*	12	None found	As above
	BRCA			
	*ESCA*			
	HNSC			
	*LUSC*			
	*STAD*			
	*UCEC*			
ADRB1	*ESCA*	14	None found	
ADRB2	HNSC	15	Propranolol suppresses pancreatic and breast cancers invasion, protects patients with skin melanoma from disease recurrence and death and avoids EGFR inhibitor resistance in lung cancer [40, 41, 76, 77]	Treatment of hypertension or irregular heart rate
	KIRC			
	KIRP			
	*THCA*			
CNR1	*ESCA*	1	Rimonabant inhibits human breast cancer cell proliferation [78]	Anorectic antiobesity (drug withdrawn)
	*THCA*			
HTR7	*BLCA*	20	None found	Many indications, including depression, psychosis and panic disorder

All receptors show mean expression (RPKM) values > = 100. For G_i/o_-linked GPCRs we sought only those that showed no significant fold-change when comparing tumors to wild-types (i.e., as overexpression likely indicates an oncogenic activity); for G_s_-linked GPCRs we included all that were not significantly under-expressed. Cancer types in italic are those where *GNAS* activating mutations or G_i/o_ GPCR down-regulation/deactivation is observed. Agonist/antagonist classification has been derived from IUPHAR [35]. +Fingolimod (phosphorylated metabolite) is a functional antagonist for S1PR1. It first acts as an agonist, but induces degradation of S1PR1

## Discussion

Understanding the role played by somatic mutations in cancer is critical for the interpretation of large datasets from high-throughput sequencing projects and the development of personalized therapies. Considering functional relationships, or biological context, can help identify novel driver mutations, even for less frequently mutated genes [44]. Previous studies used protein family evolutionary relationships to highlight positions significantly affected by somatic mutations [16] and suggested that sparse mutations affecting equivalent domain positions in known drivers might display similar downstream consequences [16]. Similarly, the usage of protein domain information was shown to improve the detection of deleterious variants of genetic diseases [45]. Here, we employed a similar approach to analyse non-synonymous mutations and, to further infer on their biological role, we pinpointed causal relationships by systematically analysing their mutual exclusivity along biological pathways.

Our pan-cancer network includes just 51 (5%) genes from the Cancer Gene Census. Missing genes are from protein families lacking either individual mutation-enriched positions, or mutual exclusivity. The majority of the genes are not currently in the census, and could be new (usually rare) oncodriver mutations. For example, a few zinc-finger genes have been classified as oncodrivers in the Census (e.g., *ZNF311* [46]) and there is mounting evidence for their involvement in several cancer types, most often as tumor suppressors, by regulating the transcription of genes important for tumor progression [46].

We propose that novel inhibitory mutations on G_i/o_-coupled receptors may converge to produce a similar outcome to *GNAS* activating mutations, resulting in increased cAMP signalling. Moreover, these events appear to be part of a wider dysregulation of GPCR mediated signalling in multiple cancers (Fig. 4a), which we predict to lead to *GNAS* overactivity, owing to its much higher expression than all other G-proteins (Fig. 4a, b). This is also supported by our analysis of non-synonymous mutations and copy number variants (CNVs) in genes involved in cAMP signalling (Fig. S14a). About a third of patients (9k of 28k) have such mutations, and as expected activating mutations and/or CNV gains prevail in genes increasing cAMP (i.e., Gα_s_ subunits and Adenylyl cyclases), with CNV losses being more common in genes that lower cAMP (i.e., Gα_i_ subunits and Phosphodiesterases), (Fig. S14b). Generally, many cancers evolve towards higher cAMP by a variety of different mechanisms.

Several GPCRs are involved in cancer progression, metastasis and therapy resistance [47]. GPCRs and their cognate Gα proteins are extensively mutated in cancer samples, though the functional consequences are not always clear and appear to be context dependent. For example, it is well established that *GNAS* p.R201^G.hfs2.2^ (on SWI) and *GNAQ/11* p.Q209^G.s3h2.3^ (on SWII) activating mutations are oncogenic [30]. Recent experiments have begun to decipher the functional consequences of the *GNAS* p.R201C mutation in pancreatic ductal adenocarcinomas, where it modulates *KRAS* p.G12V initiated neoplasia [48, 49]. We also found SWI mutations in *GNAI2* (p.R179), in skin melanoma and lymphomas, where it has oncogenic effects by upregulating ERK1/2 [50], and in *GNA13* (p.R200; in bladder carcinoma), which is not yet fully understood.

Functionally equivalent mutations on GPCRs have been seen to replace those in Gα proteins. For example, mutually exclusive *P2RY8*, *GNA13* and *RHOA* mutations have similar inactivating effects in B-cell derived lymphomas [51, 52]. Activating mutations of *CYSLTR2* (p.L129Q) in uveal melanoma are mutually exclusive and functionally equivalent to mutations in *GNAQ/11* (p.Q209) [53]. Compensatory mutations with opposite functional outcomes have been reported in pituitary tumors, where the effect of activating *GNAS* mutations can be replaced by inactivating mutations on G_i/o_ proteins [54] or on AIP [55].

Thus, widespread inactivating mutations in G_i/o_-linked GPCRs similarly suggests that their restraining activity on *GNAS* is lost and, as part of the tumorigenic process, cAMP signalling is persistently activated and leads to tumor progression. It has been shown that a cAMP signalling network, involving upstream GPCRs, is responsible of MAP kinase inhibitors resistance in melanomas [42]. The observation that DRY arginine mutations are associated to lower survival in melanoma, suggests that these mutation events might indeed concur to these mechanisms.

Knowledge of rare oncodrivers, including several identified here, can impact cancer diagnostics and treatment. For example, of the 26k samples across all cancers lacking common *TP53* mutations, 2.2k (7.8%) show mutations in the Zinc-finger genes and positions mentioned above, thus potentially providing a molecular mechanism for tumor suppressor activities that might have been overlooked. More tantalizingly, observation of deactivating mutations in G_i/o_ coupled GPCRs could indicate suitability for treatment of to be developed G_s_ inhibitors, or readily available inhibitors of G_s_ coupled receptors, including the recently proposed off label use of beta-blockers (e.g., propranolol) or by exploiting the wealth of agonists activating G_i/o_ coupled receptors. The search for these in the future must naturally consider the complete context of GPCR/G-protein signalling pathways in each cancer.

The synthesis of functional information with genetic variants can uncover new molecular insights into diseases, including new disease genes, and specific molecular mechanisms. This holds much promise for developing personalized diagnoses and therapies across many clinical subjects.

## Materials and methods

### Datasets

We extracted confirmed somatic, non-synonymous mutations from version 79 of COSMIC [56] genomes (http://cancer.sanger.ac.uk/cosmic). We mapped 22842 of 28089 (81%) of the associated Ensembl [57] transcripts to Uniprot [58] canonical isoforms, which left 1.5 M unique protein mutations (corresponding to a total of 4.6 M mutated alleles), from 21k unique samples from 414 different cancer types. We defined cancer types using the COSMIC classification system considering Primary tissue/Tissue sub-type1 and Primary histology/Histology sub-type1 specifications.

We used the Pfam [14] database as it has the widest coverage of sequence space and provides HMM profiles for easy alignment of query sequences to the pre-existing alignments. We identified Pfam-A families within the mapped protein sequences using HMMer [59], defining highly-conserved positions as those with one amino-acid recurring in 50% or more sequences. To identify each position within the alignment, we employed the Pfam consecutive numbering scheme and labelled them using the amino acids letter from the Hidden Markov Model (HMM) consensus sequence (highly conserved residues are upper case). For GPCRs, we additionally labelled positions through the Ballesteros/Weinstein scheme [22], using the consensus domain secondary structure from the HMM model to number residues in helix regions (see Table S20). For G-proteins, we employed the established Common Gα numbering [23].

### Variant enrichment

To define domain positions within protein family alignments enriched in variants we computed the log of the observed number of variants divided by the expected (log odds). We computed the expected number by multiplying the frequency of total alleles (4.6 alleles) in the total proteome length (>11 million amino acids) times the number of domain instances in the proteome. We assessed the statistical significance of domain position enrichments, either in individual cancer types or pan-cancer, through a one-tailed binomial test, computing the prior probability by randomly shuffling of non-synonymous protein variants from each individual across the same protein. Shuffling did not produce sufficient numbers of all possible combinations we observed to generate separate distributions to calculate observed *P*-values, but was rather used to get an expected probability to use in the binomial calculation. We corrected *P*-values through the Benjamini-Hochberg or False Discovery Rate procedure [60] to give a corresponding *Q*-value (labelled *q* in the text). For individual cancers, we retained positions having 5 observed or 2.5 expected non-synonymous variants; for pan-cancer we considered positions with 20 observed or 5 expected. For both we defined significantly enriched positions as those with log odds > = 0 and *q* < = 0.01.

These thresholds were chosen heuristically, based on our experience, to narrow down the analysis to more interesting examples and to avoid potential false positives. Indeed, using no thresholds, led to an increase in the total number of identified positions, in both specific tissues and pan-cancer and in the absolute number of dubious genes (see Table S5a), defined as those more tolerant to mutations as assessed through the RVIS approach [17]. We considered only the top 2% of the most tolerant genes ranked through the RVIS score.

To correct for domain mutation rate, we randomly shuffled mutations found at domains within positions of the same domain and the same protein. Mutations found outside domain regions, were randomly shuffled within the remaining protein sequence portion. We similarly calculated the enrichment of mutations at domain positions by using this alternative background model.

### Mutual exclusivity analysis of functionally related protein positions

We defined functionally interacting genes based on their membership to a subset of Reactome (http://reactome.org/) pathways [18], defined according to size criteria to represent specific functional units. We considered pathways with fewer than 200 proteins from the *FrontPageItem* list. Sub-pathways were included if their size was less than 300. For sub-pathways with >300 proteins, we considered their sub-pathways. The threshold for the second level is based on the assumption that the lower level pathways should be more akin to functional units than those in upper levels. This allowed us to define a set of *intermediate level* biological pathways, which we found to be a trade-off between coverage and lower inner redundancy.

We assessed the mutual exclusivity of domain positions in mutated family members participating to the same biological pathway either in individual cancer types or pan-cancer. We considered only significantly enriched domain positions (see previous section) mutated either in at least five unique samples for individual cancers, or 50 unique samples for the pan-cancer set. We assessed the mutual exclusivity for each domain position pair within a given pathway through a one tail Fischer’s exact test, correcting *P*-values through the FDR procedure (*Q*-values), and considering position pairs with *q* < = 0.1. We adopted a looser threshold for FDR correction, with respect to the enrichment analysis, as by default mutual exclusivity inspected through the Fisher’s exact is an extremely stringent test. For each comparison, we restricted our analysis only to unique samples having mutations to the same class of domain position (i.e., classifying mutations at either conserved or non-conserved positions within the same domain).

We evaluated the enrichment significance for domain positions pairs through a binomial test procedure similar to that used for individual positions, computing the prior probability by randomly shuffling non-synonymous protein variants within members of a particular pathway.

Repeating the same test by considering the entire set of lowest level pathways from Reactome hierarchy [15] led to a moderate decrease of identified positions with no change in the number of RVIS genes (see Table S5b).

### Assessing biomolecular consequences of mutations

We predicted functional consequences of COSMIC missense mutations using Mechismo [18] (mechismo.russelllab.org), which matches protein sequence positions to positions within three-dimensional structures and identifies sites affecting known interactions with other proteins, DNA/RNA or small-molecules. We considered medium-high confidence predictions, which include known structures or homologs with > = 30% of sequence identity for protein-protein interactions, > = 35% for protein-chemicals and > = 41% for protein-DNA/RNA interactions (as defined by Mechismo based on a benchmark for the accuracy of perturbed interfaces). We considered any interaction evidence, including interactions from other species, or those coming from indirect experiments (e.g., affinity purifications or co-expression).

### Analysis of transcription factor targets

We retrieved a list of transcription factors putative target genes from an R package (https://github.com/slowkow/tftargets), which is a collection of several gene regulatory network experiments (including Chip-Seq data [61–66]). We then assessed the pairwise similarity of transcription factors target genes with a Jaccard score.

### Oncodriver co-occurrence and survival time analysis

For each retained domain position, we assessed the co-occurrence of mutations of known oncodrivers from the Cancer Genes Census (CGC) [1]. We considered simple non-synonymous mutations, copy number variants (CNVs) and structural rearrangements for a total of 24k unique samples, corresponding to the 82% of the total.

We considered separately oncogenes and tumor suppressor genes (TSG), and defined a third category, Signalling Oncodrivers, which we generated by mapping all CGC genes to the Reactome “Signal Transduction” (top hierarchy) pathway. We then manually check this list based on literature inspection to include additional candidates and/or remove genes with no role in signal transduction. Mutual exclusivity between domain positions and oncodriver alterations was done through a one tailed Fisher’s exact test, correcting *P*-values through the FDR procedure (*Q*-values), considering as significant those instances having a *q* < 0.1.

We collected information for 17323 donors from release 23 of ICGC (icgc.org), and matched these to the corresponding COSMIC sample. We considered vital status (alive/deceased), disease status (complete remission or not – i.e., partial remission, relapse, progression) and survival time. We used the LogRank test to identify patient groups affected by particular domain positional mutations and having a statistically significant (*P* < 0.05) difference in survival time. We generated Kaplan-Meier survival analysis plots for the most significant examples. We employed Cox’s proportional hazard model to predict hazard ratios and survival probability of patients affected by interface-perturbing mutations, employing age, sex and cancer type as covariates.

For all the clustering and statistical analysis we used python (www.python.org/) through scipy (www.scipy.org/), statsmodels (statsmodels.sourceforge.net/) and lifelines (lifelines.readthedocs.org/en/latest/) libraries.

### TGFα shedding assay

Transforming growth factor-α (TGFα) shedding assay was performed as described previously [34] with minor modifications. HEK293A cells were seeded in a 6-well culture plate (Greiner Bio-One) at a density of 2 × 10^5^ cells per well, with 2 ml of complete DMEM and cultured for 1 day. The cells were transfected with a mixture of plasmids encoding a codon-optimized alkaline phosphatase (AP)-tagged TGFα (AP-TGFα, 1 µg, pCAGGS vector) and a GPCR of interest (200 ng) combined with 5 µl of 10 µl of 1 mg ml^−1^ PEI solution and 95 µl of Opti-MEM® I Reduced Serum Medium. For some GPCRs, a plasmid encoding a chimeric Gα_q/s_ subunit (100 ng, pCAGGS vector) was co-transfected. After 1-day incubation, the cells were trypsinized with 0.05% trypsin- and 0.53 mM EDTA-containing D-PBS, neutralized with the complete DMEM, collected in a 15-ml tube, centrifuged at 190 g for 5 min, and suspended in 6 ml of pre-warmed HEPES-HBSS. The cell suspension was left for 15-min to settle spontaneous AP-TGFα release caused by trypsinization. Afterward, the cells were centrifuged and suspended in 6 ml of HEPES-HBSS and seeded in a 96-well culture microplate (Greiner Bio-one) at a volume of 80 μl per well. The cell plate was placed in a CO_2_ incubator for 30 min and mixed with 10 µl of 10X titrated test compounds diluted in 0.01% BSA-containing HEPES-HBSS. After 1-h incubation, the cell plate was centrifuged at 190 g for 2 min, and 80 µl of conditioned media was transferred to an empty 96-well plate (conditioned media (CM) plate). AP reaction solution (10 mM *p*-nitrophenylphosphate (*p*-NPP, disodium salt, Wako Pure Chemicals), 120 mM Tris–HCl (pH 9.5), 40 mM NaCl, and 10 mM MgCl_2_) was dispensed into the cell plate and the CM plate at a volume of 80 µl per well. Absorbance at a wavelength of 405 nm were measured using a microplate reader (SpectraMax 340 PC384, Molecular Devices), before and after a 1 h incubation at room temperature. For each well measurement, change in the absorbance unit (Abs_405_) during 1 h incubation with *p*-NPP solution (∆Abs_405_) was used as a relative amount of AP-TGFα. Relative AP-TGFα in the CM plate was calculated by dividing ∆Abs405 in the CM plate by the total (the CM plate and the cell plate) ∆Abs_405_, followed by multiplication by a factor of 1.25 (80 µl transferred volume out of total 100 µl). Compound-induced AP-TGFα release was obtained by subtracting spontaneous AP-TGFα release (e.g., vehicle-treated relative AP-TGFα in the CM plate). The AP-TGFα release was fitted to a four-parameter sigmoidal concentration-response curve (Prism 7 software, GraphPad Prism).

### cAMP Glosensor assay

An in-house-modified cAMP Glosensor^TM^ assay was performed as follows. HEK293A cells (Thermo Fisher Scientific) were seeded in a 6-cm culture dish (Greiner Bio-One) at a density of 2 × 10^5^ cells per well, with 4 ml of Dulbecco’s Modified Eagle Medium (DMEM), supplemented with 10% fetal calf serum (FCS) and 100 U ml^−1^ penicillin and 100 µg ml^−1^ streptomycin (complete DMEM), and cultured for 1 day. Transfection solution was prepared by combining 190 µl of Opti-MEM® I Reduced Serum Medium (Thermo Fisher Scientific), plasmids encoding pGlo-22F (2 µg, codon-optimized for human cell expression, Genscript, pCAGGS vector) and a GPCR of interest (400 ng, pCAGGS vector or pcDNA3.1 vector), and 10 µl of 1 mg ml^−1^ PEI solution (Polyethylenimine “Max”, (Mw 40,000), Polysciences). The resulting transfection solution was added to the cells. After 1-day culture, the transfected cells were detached with 1 ml of 0.53 mM EDTA-containing Dulbecco’s Phosphate-Buffered Saline (D-PBS), mixed with 2 ml of Hank’s Balanced Salt Solution (HBSS) containing 5 mM HEPES (pH 7.4) (HEPES-HBSS) and centrifuged at 190 g for 5 min. The cell pellet was suspended in 1.2 ml of 0.01% (w/v) bovine serum albumin (BSA, fatty-acid-free and protease-free grade, Serva)-containing HEPES-HBSS and the cell suspension was seeded in a 96-well half-are white microplate (Advanced TC, Greiner Bio-One) at a volume of 30 µl per well. The cells were loaded with D-luciferin (Wako Pure chemicals) at 10 µl of 8 mM solution and incubated at room temperature for 2 h in dark. After measurement of initial luminescent signals with a microplate luminometer (SpectraMax L, Molecular Devices), 10 µl of 5X titrated test compound diluted in 5 µM forskolin were manually added to the cell and incubated at room temperature for 10 min. Afterward, luminescent signals were measured and normalized to the initial counts. The luminescent signals were fitted to a four-parameter sigmoidal concentration-response curve using the Prism 7 software (GraphPad Prism) and the values for pEC_50_ (equal to −Log_10_ EC_50_ [M]) and *Emax* were calculated from the curve.

### Differential expression analysis

We obtained raw read counts for each gene in all cancer types released from TCGA (The Cancer Genome Atlas) before 26th February 2016. To avoid unreliable results, we selected 16 cancer types with at least 10 pairs of tumor-normal tissue matches. We used Deseq2 [67] for differential gene expression analysis in each cancer type and adjusted *p*-values (*padj*) for multiple testing by the Benjamini & Hochberg method [60]. We considered only values with corrected *p*-values (*padj* or *q*) < 0.01.

We also performed differential expression analysis of samples carrying GPCR R^3.50^ or G-protein known activating mutations (i.e., on position G.hfs2.2 on SWI, corresponding to *GNAS* p.R201, and on G.s3h2.3 on SWII, corresponding to GNAQ/11 p.Q209). For both, we excluded from the control both normal tissue samples as well as samples carrying mutations of the second class (either GPCR or G-protein) to be compared.

We considered the top 3 TCGA cancer types carrying 7 M R^3.50^ mutations, i.e., skin melanoma (SKCM: 19 samples), uterus endometrial carcinoma (UCEC: 11 samples) and stomach adenocarcinoma (STAD: 10 samples). Only significant genes (*q* < 0.01) with an absolute Log Fold Change (LFC) > = 2 were retained. Gene enrichment analysis of the overlapping genes between 7TM R^3.50^ and Gα activating mutations in melanoma was performed through g:Profiler [68] and visualized through Enrichment map [69].

### Integrative analysis of GPCR’s activity in cancer

We grouped GPCRs based on their coupling preferences by using primary and secondary coupling information available from the literature (http://www.guidetopharmacology.org/) [35]. We then estimated the activity for each G-protein family by combining differential expression data for G-protein and GPCRs according to available coupling information. We considered the following G-protein groups: G_s_ (*GNAS, GNAL*), G_i/o_ (*GNAI1, GNAI2, GNAI3 GNAO1*), G_q/11_ (*GNAQ, GNA14, GNA15*) and G_12/13_ (*GNA12, GNA13*).

We estimated the activity (A_G_) of each G-protein family, in a given cancer type, according to the following equation:

1 $$A_G = \mathop {\sum }\limits_1^n m_{GPROT}^s \ast m_{GPCR}^s \ast c$$

where, *m* is the base mean of normalized counts for all samples (i.e., cancer and control) used for differential expression analysis, normalized for sequencing depth; *s* is a scaling factor corresponding to the Log Fold Change (LFC), when significant (i.e., *padj* < 0.01), alternatively it’s set to 1;

*c* is a coupling constant set to 1 if the coupling is reported in IUPHAR, elsewhere is 0; *n* is the number of G-protein for a given family.

For GPCRs, we calculated the fraction of samples containing likely deleterious mutations, i.e., stop gains, frame-shift insertions/deletions or non-synonymous mutations affecting highly conserved 7TM positions. For G-proteins, we calculated the fraction of samples affected by known specific oncogenic mutations.

The column of the matrix in Fig. 4a as well as the top dendrogram were derived from hierarchical, complete-linkage clustering of GPCR coupling group significant LFCs.

## Supplementary information

Supplemental material legends

Supplementary Tables

Supplementary Figures

Figure_S12

Figure_S13

Figure_S14

## Data Availability

Code and datasets to reproduce the analysis available at: http://www.russelllab.org/multigene-oncodriver.
