# Supplementary material for: Rare, functional, somatic variants in gene families linked to cancer genes: GPCR signaling as a paradigm
Source: Oncogene. 2019 Jul 23;38(38):6491–506. doi: 10.1038/s41388-019-0895-2 (PMC6756116; doi:10.1038/s41388-019-0895-2)
Supplement: Supplementary file 1 — Supplemental material legends [file 41388_2019_895_MOESM1_ESM.docx]

# Supplementary Information

# Supplementary Tables

**Table S1**: domain position mutational enrichment (pancancer;Binomial Q-val<0.1)

**Table S2**: domain position mutational enrichment (tissue-level analysis; Binomial Q-val<0.1)

**Table S3**: domain hotspot mutual exclusivity within pathways (Pancancer; Fischer's and Binomial Q-val<0.1)

**Table S4**: domain hotspot mutual exclusivity within pathways (Tissue-level analysis; Fisher's and Binomial Q-val <0.1)

**Table S5a:** domain hotspot enrichement analysis varying parameters (mutation cutoff) and background model

**Table S5b:** domain hotspot mutual exclusivity analysis varying parameters (mutation cutoff and pathway dataset)

**Table S6**: conserved position in domains with significantly mutated positions

**Table S7**: common mechismo predicted effect of mutually exclusive domain hotspots (Pancancer)

**Table S8**: overlap (jaccard score) of downstream target genes of transcriptional regulatory genes found mutually exclusively mutated

**Table S9**: TP53 and zinc-fingers domain hotspot mutual exclusivity within pathways (Tissue-level; Fischer's and Binomial Q-val<0.1)

**Table S10** Cancer somatic missense mutations at 7TM domain positions

**Table S11**: Cancer somatic missense mutations at G-alpha domain positions

**Table S12**: Survival analysis (Cox's proportional hazard models) for significant domain hotspots (pancancer)

**Table S13**: Survival analysis (Cox's proportional hazard models) for significant domain hotspots (tissue-level analysis)

**Table S14**: Survival analysis (Cox's proportional hazard models) for significant domain hotspots (cancer type-level analysis)

**Table S15**: Census genes participating to Reactome's "Signal transduction" pathways are labelled as "Signaling Oncodriver"

**Table S16**: mutual exclusivity analysis of signaling oncodrivers and domain hotspots mutations (cancer type-level analysis)

**Table S17**: Parameters for the TGFα shedding assay

**Table S18**: Parameters for the cAMP assay

**Table S19**: differential expression in 16 TCGA cancer types of GPCRs with coupling and approved drugs information

**Table S20:** PFAM 7tm_1 domain consensus / Ballesteros-Weinstein numbering correspondences

# Supplementary figure legends

**Figure S1:** protein families with domain positions most significantly affected by mutations: for each family, the median of q values of significantly perturbed positions (FDR < 0.1) is shown. The x-axes and y-axes show the values obtained from pancancer- and tissue-level analyses. Dots are coloured (from bright to dark red) proportional to the number of significant positions, while the diameter is proportional to the total number of unique samples with mutations at these domain positions. Genes with significantly perturbed interfaces (median of q-values  <= 10^-7^ in either pancancer- or tissue-level analyses) are labelled in dark gray. For space reasons the following significantly perturbed families are not shown: PUFD, DUF2431, DOR, NAD_binding_2, Arfaptin, SNARE_assoc, TORC_C, JCAD, Xan_ur_permease, CKAP2_C, Xylo_C, DUF846, TMEM37, DUF3454, RGM_C, Choline_transpo, Ist1, Arv1, RBM39linker, L6_membrane, AJAP1_PANP_C, Fumble, Xpo1, ecTbetaR2, IF-2B, Dynamitin, Helicase_C_4, Tmemb_cc2, 2-oxogl_dehyd_N, FAM75, MutS_II, CLCA, DUF3736, Tmemb_185A, RB_B, CDC27, DUF1907, Myc_N, FRG2, Nramp, Med15, PDEase_I_N, CENP-N, DNA_pol_B_exo1, ALS2CR11, VWA_2, GREB1, PI3Ka.

**Figure S2:** a) stacked bar representation of the total number of significantly mutated domain positions for each Reactome pathway in the pan-cancer set. Colours are domain specific. b) Pie chart representation of the distribution of significant domain positions in each cancer tissue. Domain colours are as in a).

**Figure S3:** 2D representation of common perturbed interactions predicted for mutations at significant domain positions. Colours are specific to the class of the interaction, i.e. protein (PPI), chemical (PCI), nucleic acids (PDI) or mixed.

**Figure S4:** Kaplan-Meier curve for patients affected by mutations at GPCR (PFAM 7tm_1) position R97^4.40^ in the pan-cancer set.

**Figure S5:** 2D representation of patients (columns) affected by Class A GPCR (non olfactory) and G-alpha mutations at either Gα SWI arginine (dodgerblue) or GPCR R82^3.50^ (orange) in five major cancer types. In each matrix, the right panel shows GPCRs coupling preferences from IUPHAR (maroon and red colors indicate primary and secondary coupling respectively). The lower panel shows co-occurring mutations for the top 10 most mutated signalling oncodrivers

**Figure S6:** comparison of 7TM domain mutation spectra between cancer and healthy individuals: standard deviations above the average allele counts for non-synonymous mutations affecting the 7TM domain (PFAM 7tm_1) of class A GPCRs (excluding Olfactory receptors). Somatic variation from cancer genomes (Cosmic; top red histograms) are compared against the germline of healthy individuals (1000 Genomes; bottom grey).

**Figure S7:** network representation of mutually exclusive domain positions in pancreatic cancers: labels and nodes indicate domains, with the diameter of the latter being proportional to the number of unique samples with domain position mutations. Node colour (from white to dark olive green) is proportional to the number of family members mutated at a given domain position. Links represent mutual exclusive positions.

**Figure S8:** TGFα shedding assay (with a chimeric Gq/s): HEk293 cells transfected with plasmids encoding an AP-TGFα reporter construct (Glo-22F), a chimeric Gα(q/s) subunit and a test GPCR were seeded in a 96-well and stimulated with a test ligand for 1 h.

**Figure S9:** Baseline cAMP levels in cells expressing WT GPCR or DRY-mutant GPCR. HEK293 cells transiently expressing a cAMP luminescent biosensor together with a WT-GPCR or a DRY (R3.50)-mutant GPCR were loaded with D-luciferin and luminescent signal was measured. Data are shown as mean ± SEM (*n* = 4-7). *, *P* < 0.05; **, *P* < 0.01; ***, *P* < 0.001 compared with WT (t-test for GPCR with single mutant; one-way ANOVA with post-hoc Dunnett’s test for GPCR with multiple mutants).

**Figure S10:** G_s_ (lower panel )and G_s_PCR (upper panel) differential expression analysis in 16 different TCGA tumor types.

Bubble diameter is proportional to the RPKM base mean levels (i.e. considering both tumor and normal matched sample). Genes with significant (adjusted P value < 0.01) log fold change (LFC) are coloured with a scale from blue to red to indicate respectively under- and over-expression. Those with no significant LFC are grey (only genes with RPKM >= 50 in at least one cancer type are shown).

**Figure S11:** G_i/o_ (lower panel )and G_i/o_PCR (upper panel) differential expression analysis in 16 different TCGA tumor types. Figure characteristics are as in Fig. S10.

**Figure S12:** differential expression analysis of 7TM R^3.50^ mutated samples: a) network visualization of gene enrichment analysis for common genes differentially expressed in patients with 7TM R^3.50^ and Gα activating mutations in skin melanoma; b) volcano plots for differential expression analysis of 7TM R^3.50^ mutated samples in three different cancer types (i.e. skin, uterus, stomach). In each cancer type, significantly altered genes are highlighted in red and those overlapping are labelled.

**Figure S13:** integrative analysis of GPCR expression and approved drugs: bubble matrix representations of differential expression analysis of G_i/o_ (top) and G_s_ (bottom) coupled receptors in 16 different TCGA tumor types.

Bubble diameter is proportional to the RPKM base mean levels (i.e. considering both tumor and normal matched sample). Genes with significant (adjusted P value < 0.01) log fold change (LFC) are coloured with a scale from blue to red to indicate respectively under- and over-expression. Those with no significant LFC are grey. Columns and rows are arranged according to the hierarchical full linkage clustering (indicated by dendrograms) of the distances calculated based on LFC profiles. For each GPCR the full spectrum of coupling specificities (from IUPHAR) is indicated in a bottom matrix, with darker and lighter blues indicating respectively primary and secondary couplings. Information about approved agonists (up triangles) and antagonists (down triangles) – from IUPHAR- is also annotated below corresponding matrices cells.

**Figure S14**: mutation analysis of genes involved in cAMP signalling pathways, i.e. Gs and Gi/o proteins, Adenylyl cyclases, Phosphodiesterases, PKAs, and AKAPs members. a) simple mutations (black), CNV gains (green) and losses (red) are shown for each patients; b) mutation events normalized for the number of protein with stimulatory (i.e. GNAS, GNAL, ADCY1, ADCY2, ADCY3, ADCY4, ADCY5, ADCY6, ADCY7, ADCY8, ADCY9, ADCY10) or inhibitory (i.e. PDE4A, PDE4B, PDE4C, PDE4D, GNAI1, GNAI2, GNAI3) function towards cAMP levels.
