# Supplementary material for: Rare, functional, somatic variants in gene families linked to cancer genes: GPCR signaling as a paradigm
Source: Oncogene. 2019 Jul 23;38(38):6491–506. doi: 10.1038/s41388-019-0895-2 (PMC6756116; doi:10.1038/s41388-019-0895-2)
Supplement: Supplementary file 3 — Supplementary Figures [file 41388_2019_895_MOESM3_ESM.pdf]

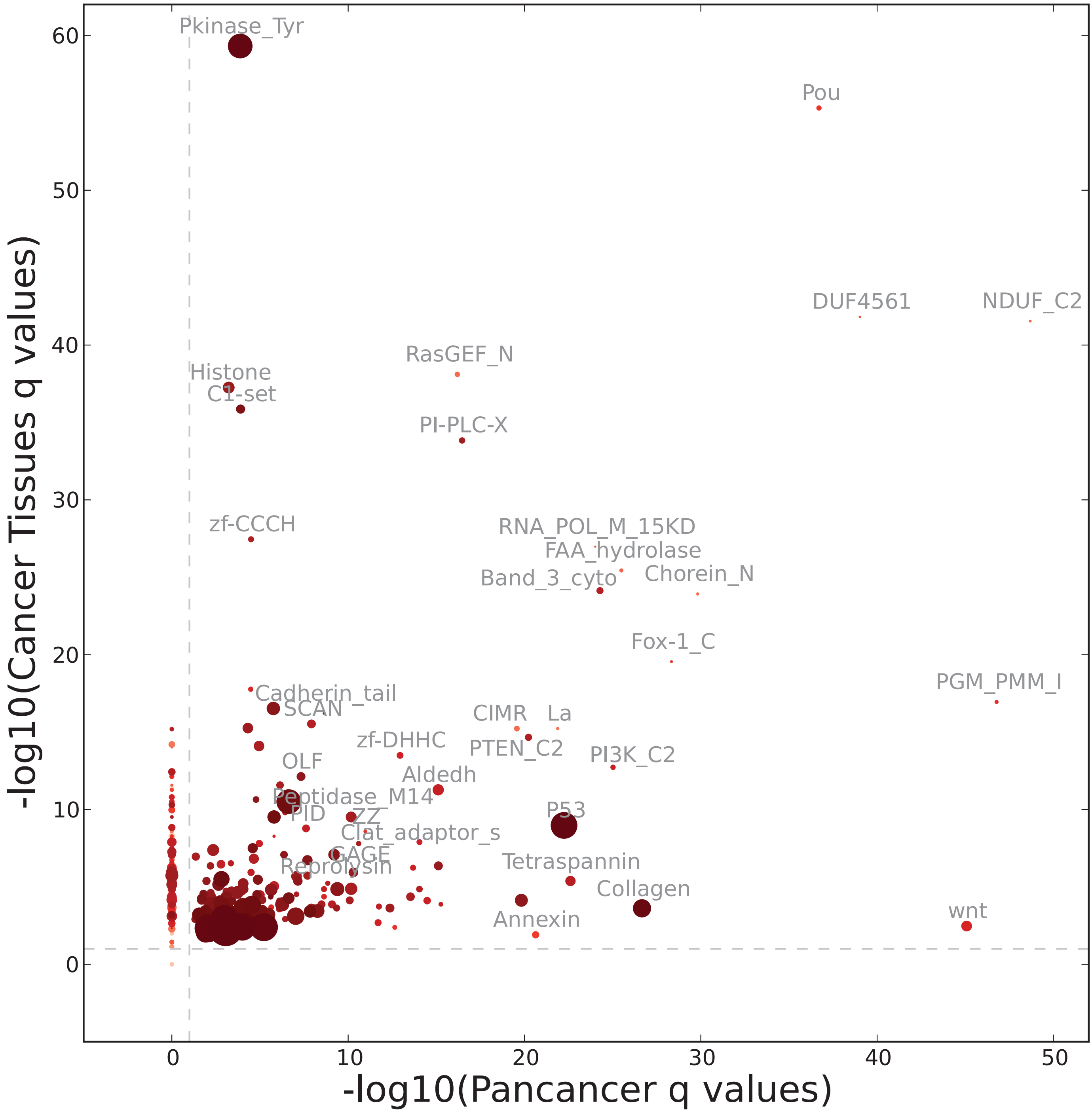

Pkinase_Tyr
Pou
DUF4561
NDUF_C2
RasGEF_N
Histone
C1-set
PI-PLC-X
zf-CCCH
RNA_POL_M_15KD
FAA_hydrolase
Band_3_cyto
Chorein_N
Fox-1_C
PGM_PMM_I
Cadherin_tail
SCAN
CIMR
La
zf-DHHC
PTEN_C2
PI3K_C2
OLF
Aldedh
Peptidase_M14
PID
ZZ
P53
Clat_adaptor_s
GAGE
Reprolysin
Tetraspannin
Collagen
Annexin
wnt
-log10(Cancer Tissues q values)
-log10(Pancancer q values)

Figure S1

a)

b)

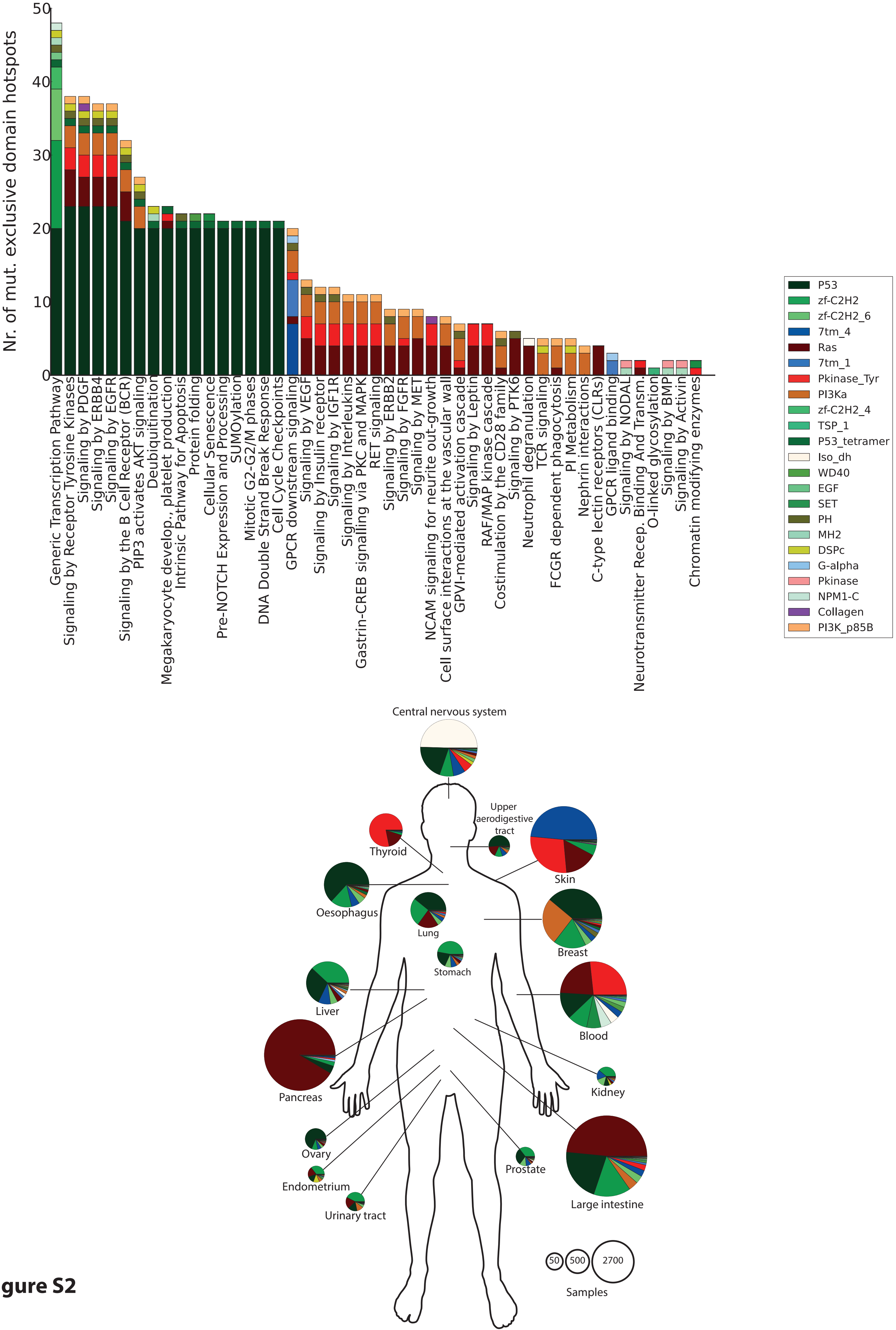
Nr. of mut. exclusive domain hotspots
0
10
20
30
40
50
Generic Transcription Pathway
Signaling by Receptor Tyrosine Kinases
Signaling by PDGF
Signaling by ERBB4
Signaling by EGFR
Signaling by the B Cell Receptor (BCR)
PIP3 activates AKT signaling
Deubiquitination
Megakaryocyte develop., platelet production
Intrinsic Pathway for Apoptosis
Protein folding
Cellular Senescence
Pre-NOTCH Expression and Processing
SUMOylation
Mitotic G2-G2/M phases
DNA Double Strand Break Response
Cell Cycle Checkpoints
GPCR downstream signaling
Signaling by VEGF
Signaling by Insulin receptor
Signaling by IGF1R
Signaling by Interleukins
Gastrin-CREB signalling via PKC and MAPK
RET signaling
Signaling by ERBB2
Signaling by FGFR
Signaling by MET
NCAM signaling for neurite out-growth
Cell surface interactions at the vascular wall
GPVI-mediated activation cascade
Signaling by Leptin
RAF/MAP kinase cascade
Costimulation by the CD28 family
Signaling by PTK6
Neutrophil degranulation
TCR signaling
FCGR dependent phagocytosis
PI Metabolism
Nephrin interactions
C-type lectin receptors (CLRs)
GPCR ligand binding
Signaling by NODAL
Neurotransmitter Recep. Binding And Transm.
O-linked glycosylation
Signaling by BMP
Signaling by Activin
Chromatin modifying enzymes
P53
zf-C2H2
zf-C2H2_6
7tm_4
Ras
7tm_1
Pkinase_Tyr
PI3Ka
zf-C2H2_4
TSP_1
P53_tetramer
Iso_dh
WD40
EGF
SET
PH
MH2
DSPc
G-alpha
Pkinase
NPM1-C
Collagen
PI3K_p85B
Central nervous system
Upper aerodigestive tract
Thyroid
Skin
Oesophagus
Lung
Breast
Stomach
Liver
Blood
Pancreas
Kidney
Ovary
Prostate
Endometrium
Large intestine
Urinary tract
50
500
2700
Samples

Figure S2

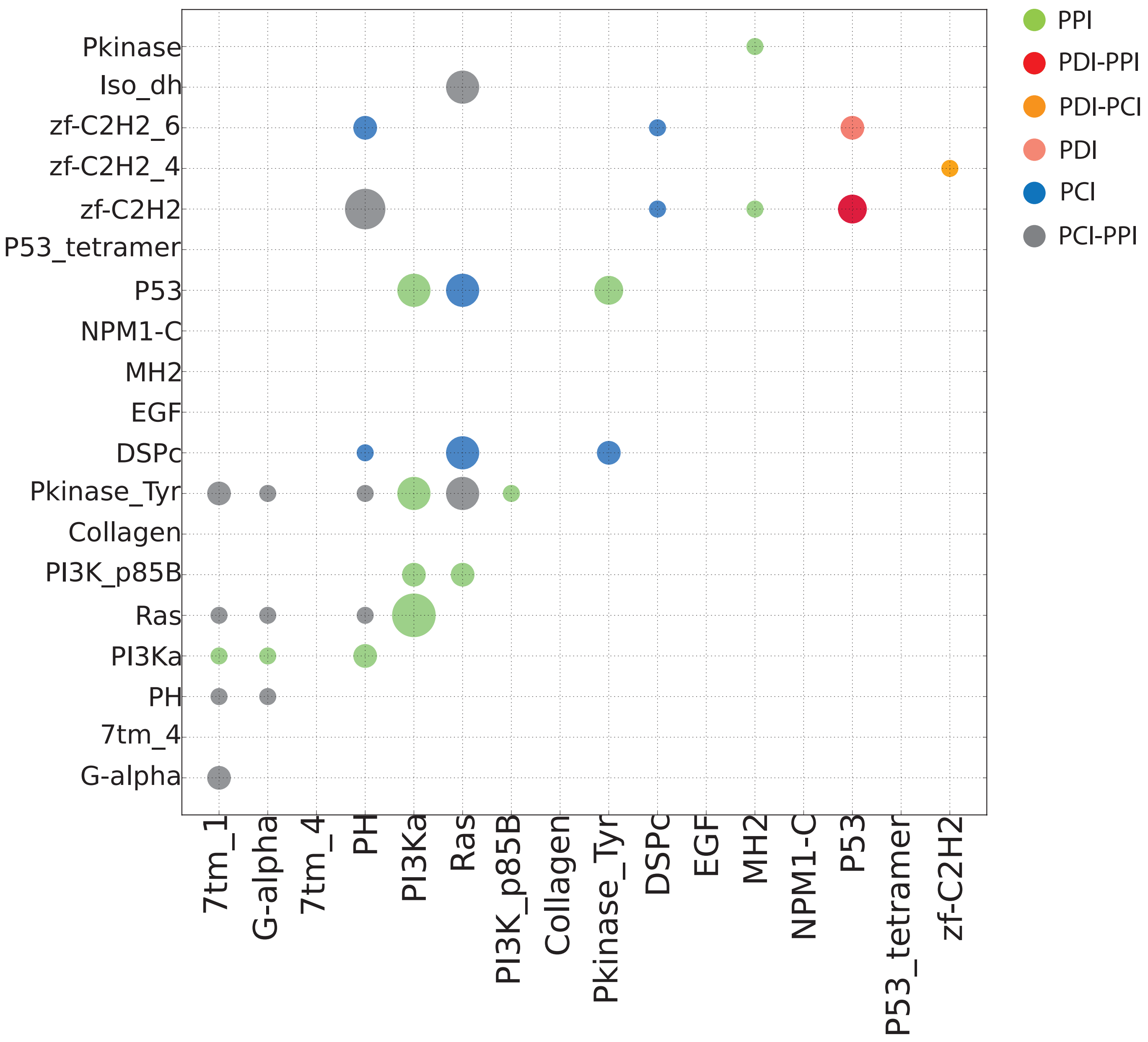

Pkinase
Iso_dh
zf-C2H2_6
zf-C2H2_4
zf-C2H2
P53_tetramer
P53
NPM1-C
MH2
EGF
DSPc
Pkinase_Tyr
Collagen
PI3K_p85B
Ras
PI3Ka
PH
7tm_4
G-alpha
7tm_1
G-alpha
7tm_4
PH
PI3Ka
Ras
PI3K_p85B
Collagen
Pkinase_Tyr
DSPc
EGF
MH2
NPM1-C
P53
P53_tetramer
zf-C2H2
PPI
PDI-PPI
PDI-PCI
PDI
PCI
PCI-PPI

Figure S3

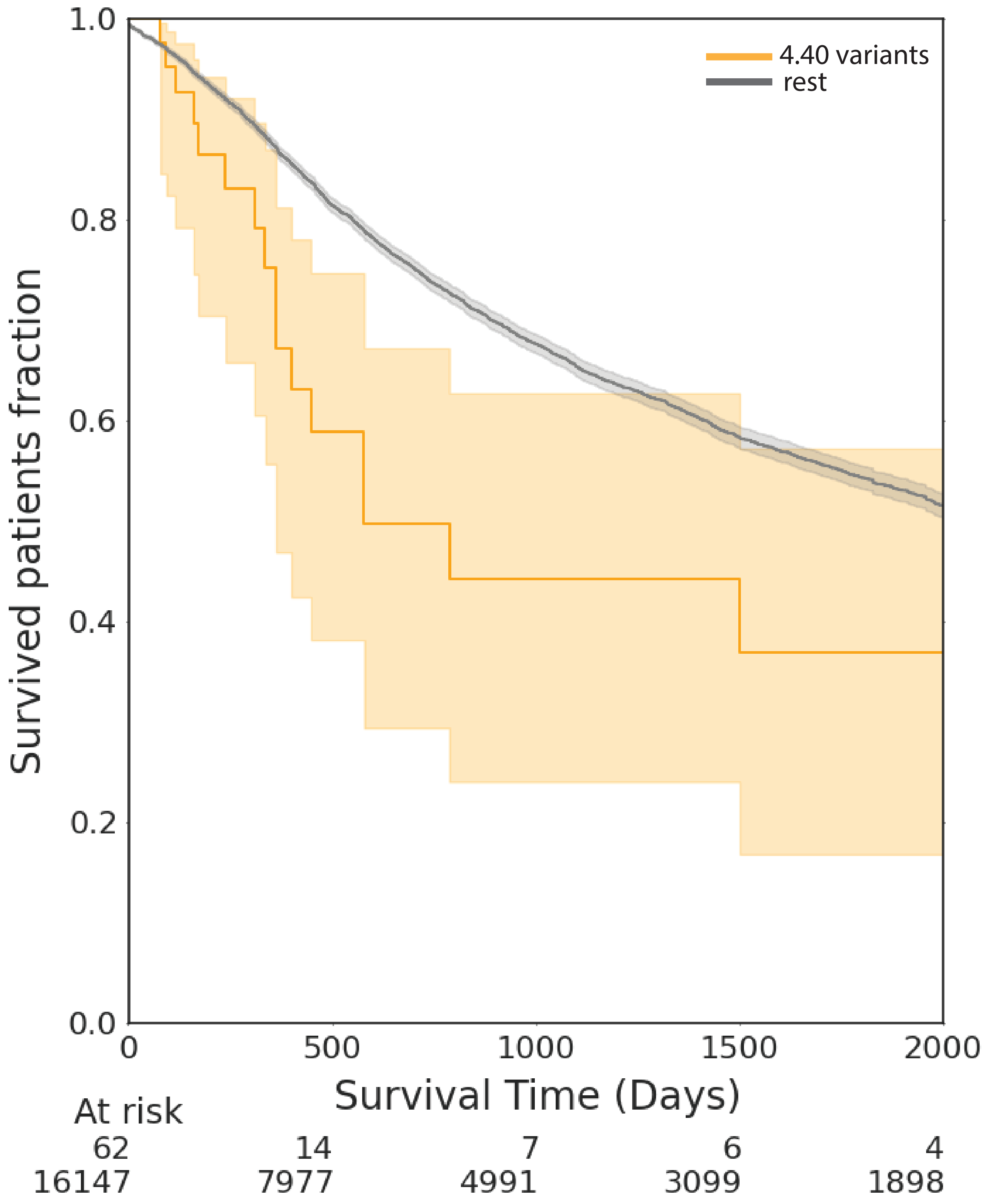

4.40 variants
rest
Survived patients fraction
1.0
0.8
0.6
0.4
0.2
0.0
0
500
1000
1500
2000
Survival Time (Days)
At risk
62
14
7
6
4
16147
7977
4991
3099
1898

Figure S4

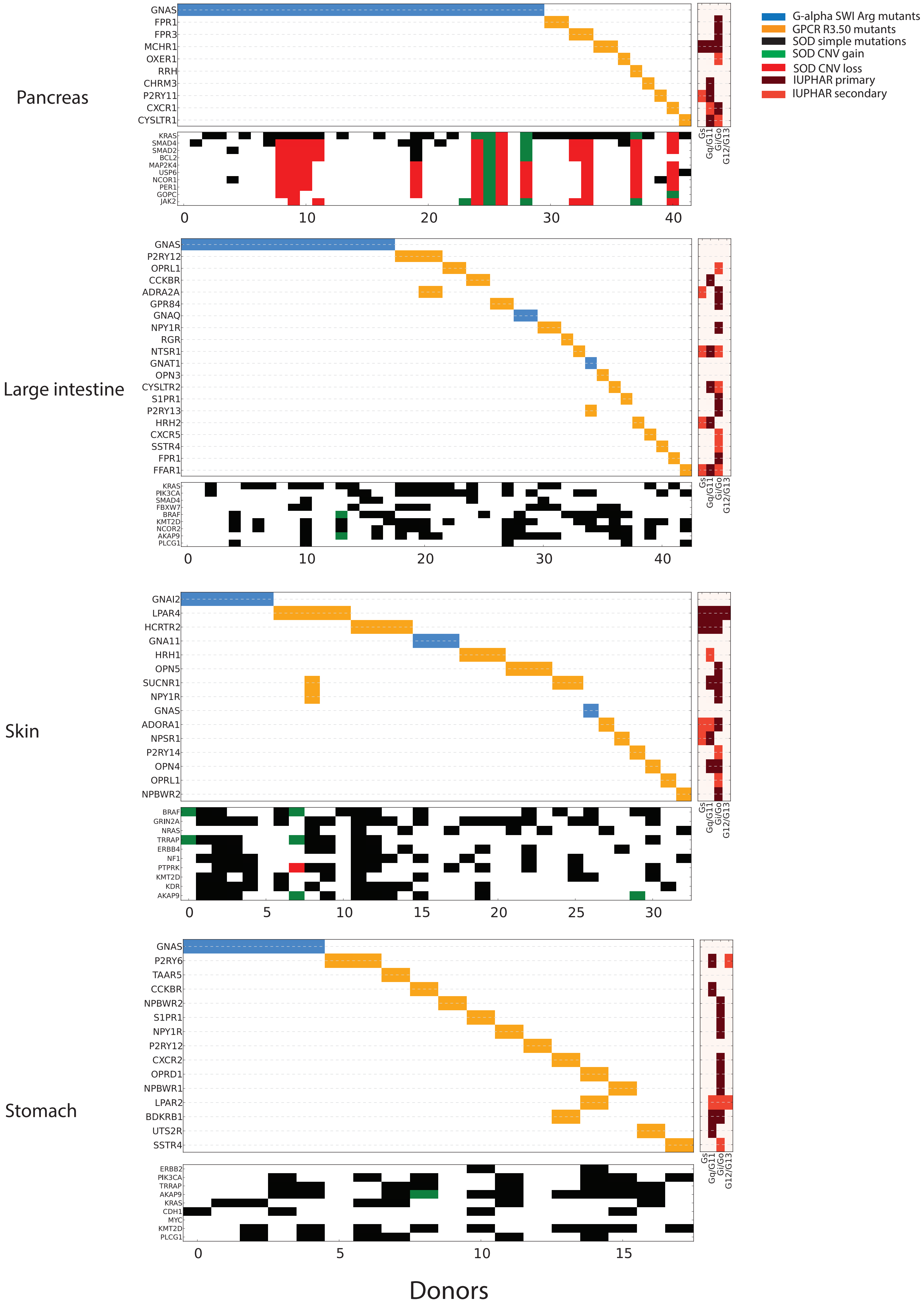

G-alpha SWI Arg mutants
GPCR R3.50 mutants
SOD simple mutations
SOD CNV gain
SOD CNV loss
IUPHAR primary
IUPHAR secondary
Pancreas
GNAS
FPR1
FPR3
MCHR1
OXER1
RRH
CHRM3
P2RY11
CXCR1
CYSLTR1
KRAS
SMAD4
SMAD2
BCL2
MAP2K4
USP6
NCOR1
PER1
GOPC
JAK2
Gs
Gq/G11
Gi/Go
G12/G13
0
10
20
30
40
Large intestine
GNAS
P2RY12
OPRL1
CCKBR
ADRA2A
GPR84
GNAQ
NPY1R
RGR
NTSR1
GNAT1
OPN3
CYSLTR2
S1PR1
P2RY13
HRH2
CXCR5
SSTR4
FPR1
FFAR1
KRAS
PIK3CA
SMAD4
FBXW7
BRAF
KMT2D
NCOR2
AKAP9
PLCG1
Skin
GNAI2
LPAR4
HCRTR2
GNA11
HRH1
OPN5
SUCNR1
NPY1R
GNAS
ADORA1
NPSR1
P2RY14
OPN4
OPRL1
NPBWR2
BRAF
GRIN2A
NRAS
TRRAP
ERBB4
NF1
PTPRK
KMT2D
KDR
AKAP9
0
5
10
15
20
25
30
Stomach
GNAS
P2RY6
TAAR5
CCKBR
NPBWR2
S1PR1
NPY1R
P2RY12
CXCR2
OPRD1
NPBWR1
LPAR2
BDKRB1
UTS2R
SSTR4
ERBB2
PIK3CA
TRRAP
AKAP9
KRAS
CDH1
MYC
KMT2D
PLCG1
Donors

Figure S5

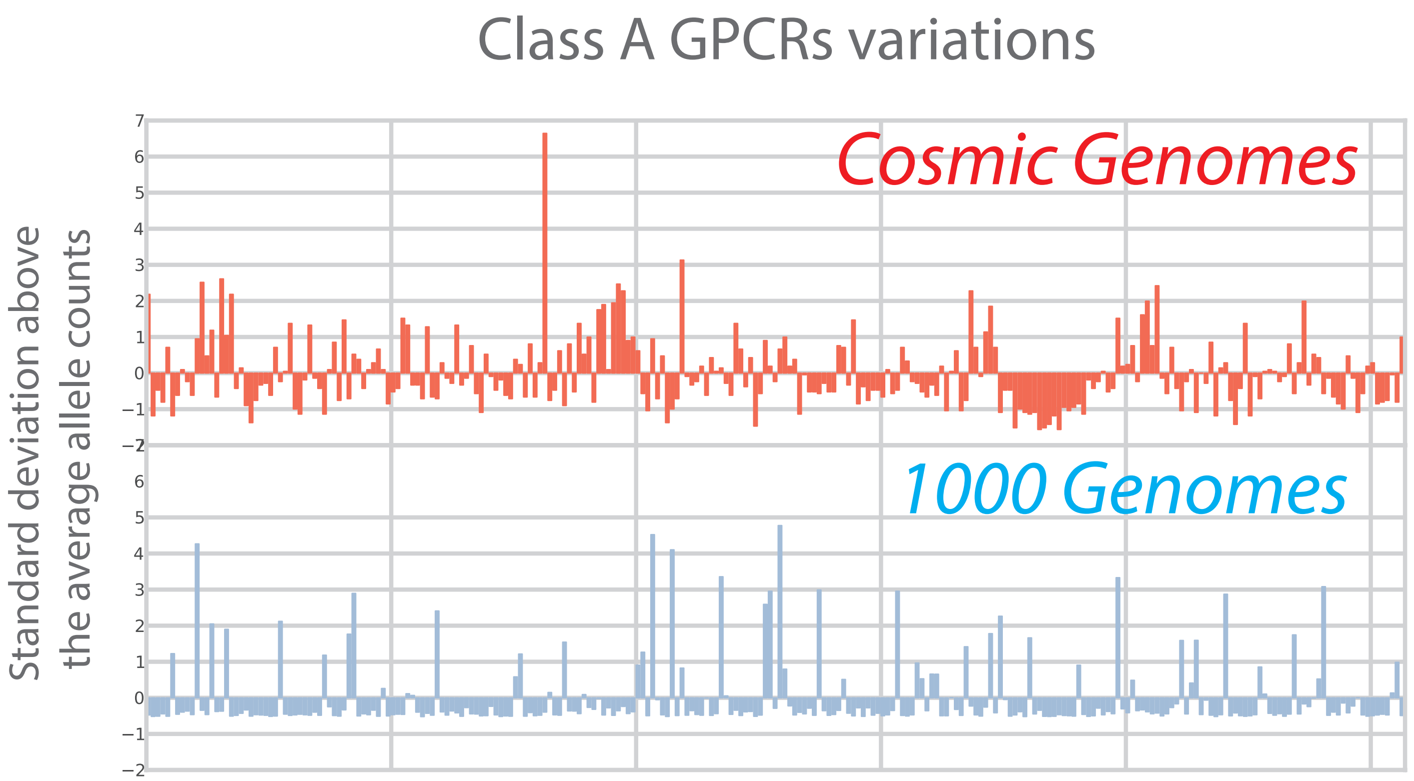
Class A GPCRs variations
Standard deviation above the average allele counts
Cosmic Genomes
1000 Genomes
7
6
5
4
3
2
1
0
−1
−2

Figure S6

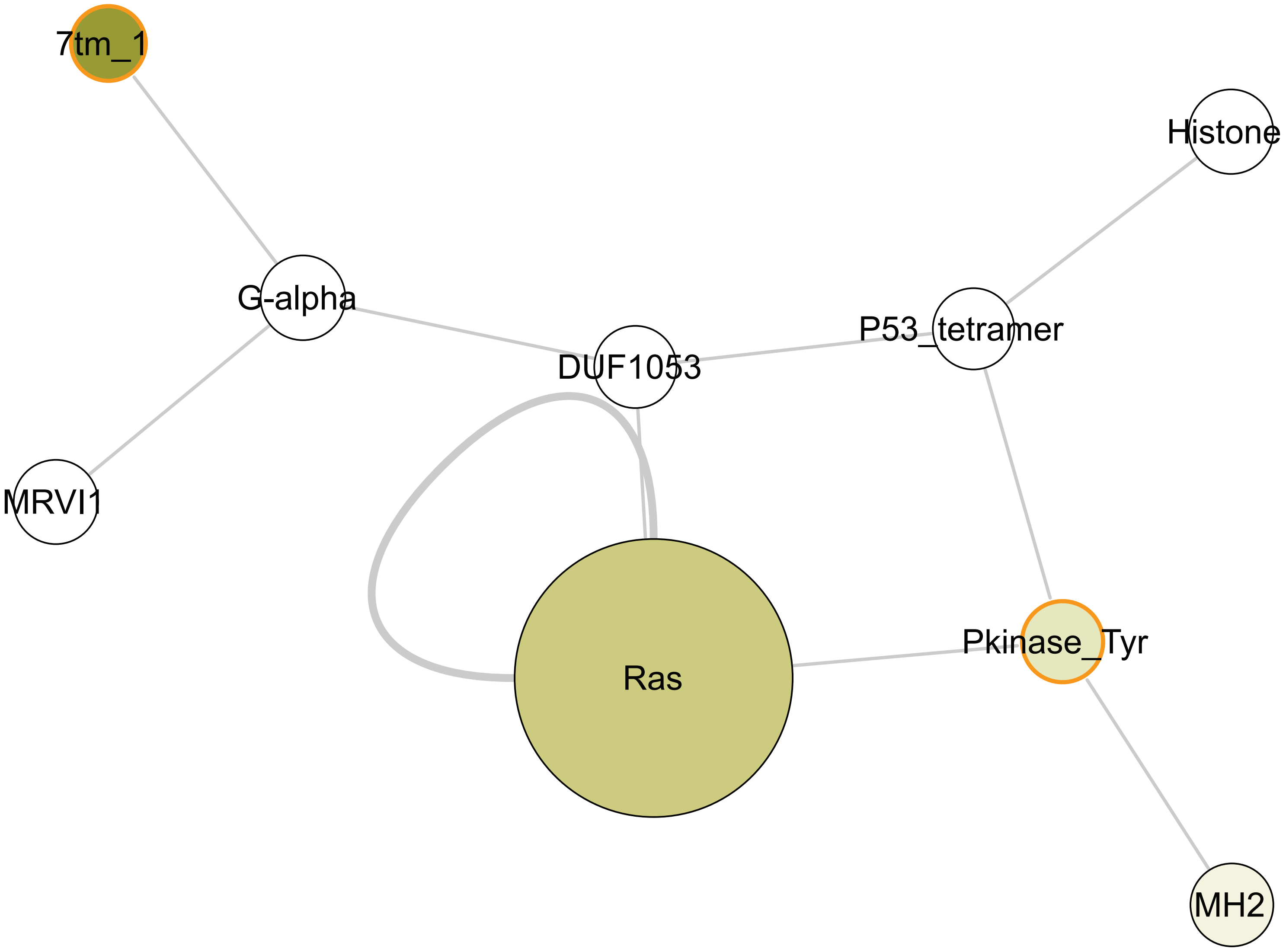
7tm_1
Histone
G-alpha
P53_tetramer
DUF1053
MRVI1
Ras
Pkinase_Tyr
MH2

Figure S7

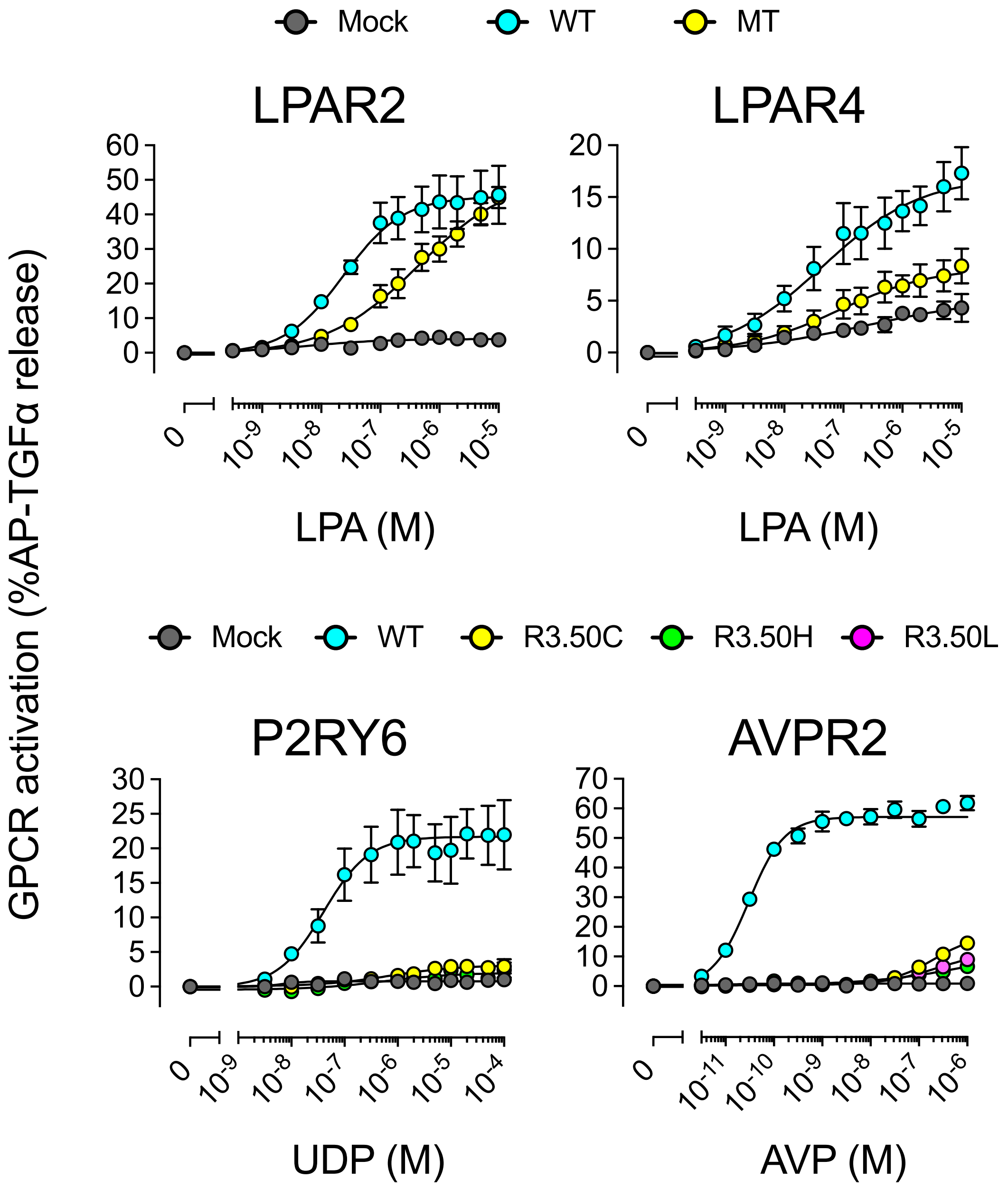

Mock
WT
MT
LPAR2
LPAR4
60
50
40
30
20
10
0
20
15
10
5
0
GPCR activation (%AP-TGFα release)
0
10^{-9}
10^{-8}
10^{-7}
10^{-6}
10^{-5}
LPA (M)
LPA (M)
Mock
WT
R3.50C
R3.50H
R3.50L
P2RY6
AVPR2
30
25
20
15
10
5
0
70
60
50
40
30
20
10
0
10^{-4}
10^{-11}
10^{-10}
UDP (M)
AVP (M)

Figure S8

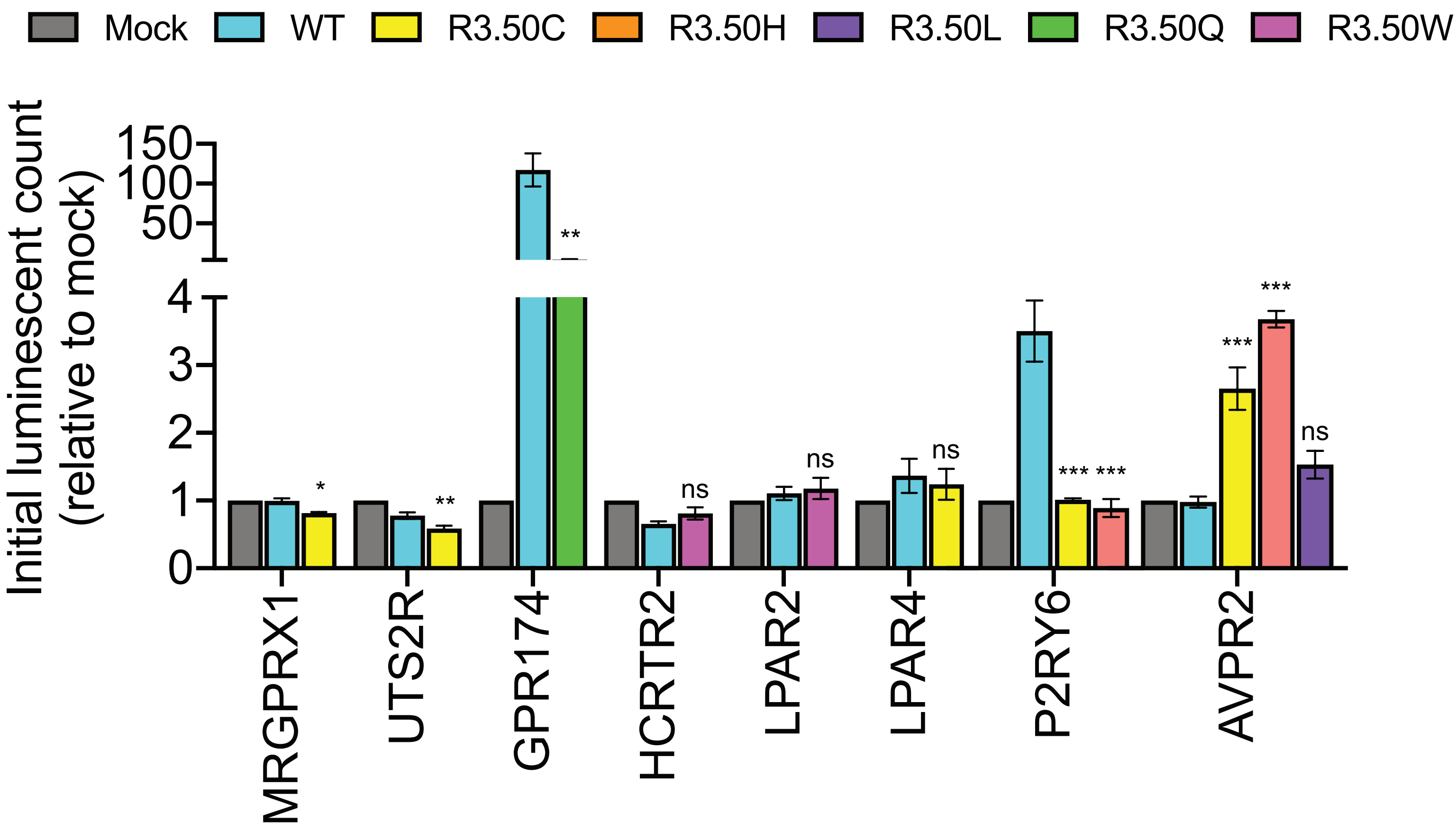
Mock
WT
R3.50C
R3.50H
R3.50L
R3.50Q
R3.50W
Initial luminescent count
(relative to mock)
150
100
50
4
3
2
1
0
**
*
**
ns
ns
ns
*** ***
***
***
ns
MRGPRX1
UTS2R
GPR174
HCRTR2
LPAR2
LPAR4
P2RY6
AVPR2

Figure S9

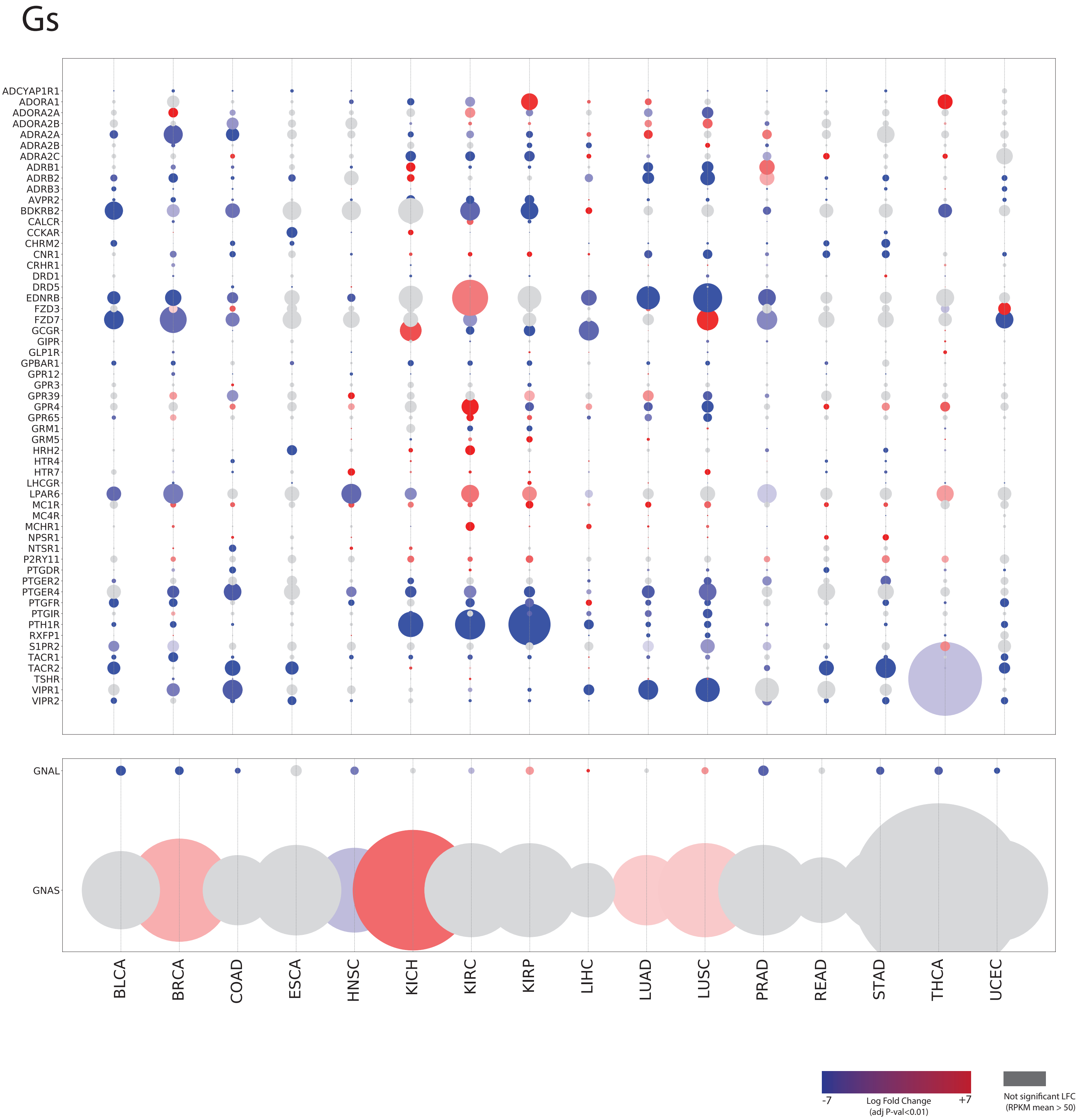
Gs
ADCYAP1R1
ADORA1
ADORA2A
ADORA2B
ADRA2A
ADRA2B
ADRA2C
ADRB1
ADRB2
ADRB3
AVPR2
BDKRB2
CALCR
CCKAR
CHRM2
CNR1
CRHR1
DRD1
DRD5
EDNRB
FZD3
FZD7
GCGR
GIPR
GLP1R
GPBAR1
GPR12
GPR3
GPR39
GPR4
GPR65
GRM1
GRM5
HRH2
HTR4
HTR7
LHCGR
LPAR6
MC1R
MC4R
MCHR1
NPSR1
NTSR1
P2RY11
PTGDR
PTGER2
PTGER4
PTGFR
PTGIR
PTH1R
RXFP1
S1PR2
TACR1
TACR2
TSHR
VIPR1
VIPR2
GNAL
GNAS
BLCA
BRCA
COAD
ESCA
HNSC
KICH
KIRC
KIRP
LIHC
LUAD
LUSC
PRAD
READ
STAD
THCA
UCEC
-7
Log Fold Change
(adj P-val<0.01)
+7
Not significant LFC
(RPKM mean > 50)

Figure S10

Gi/o

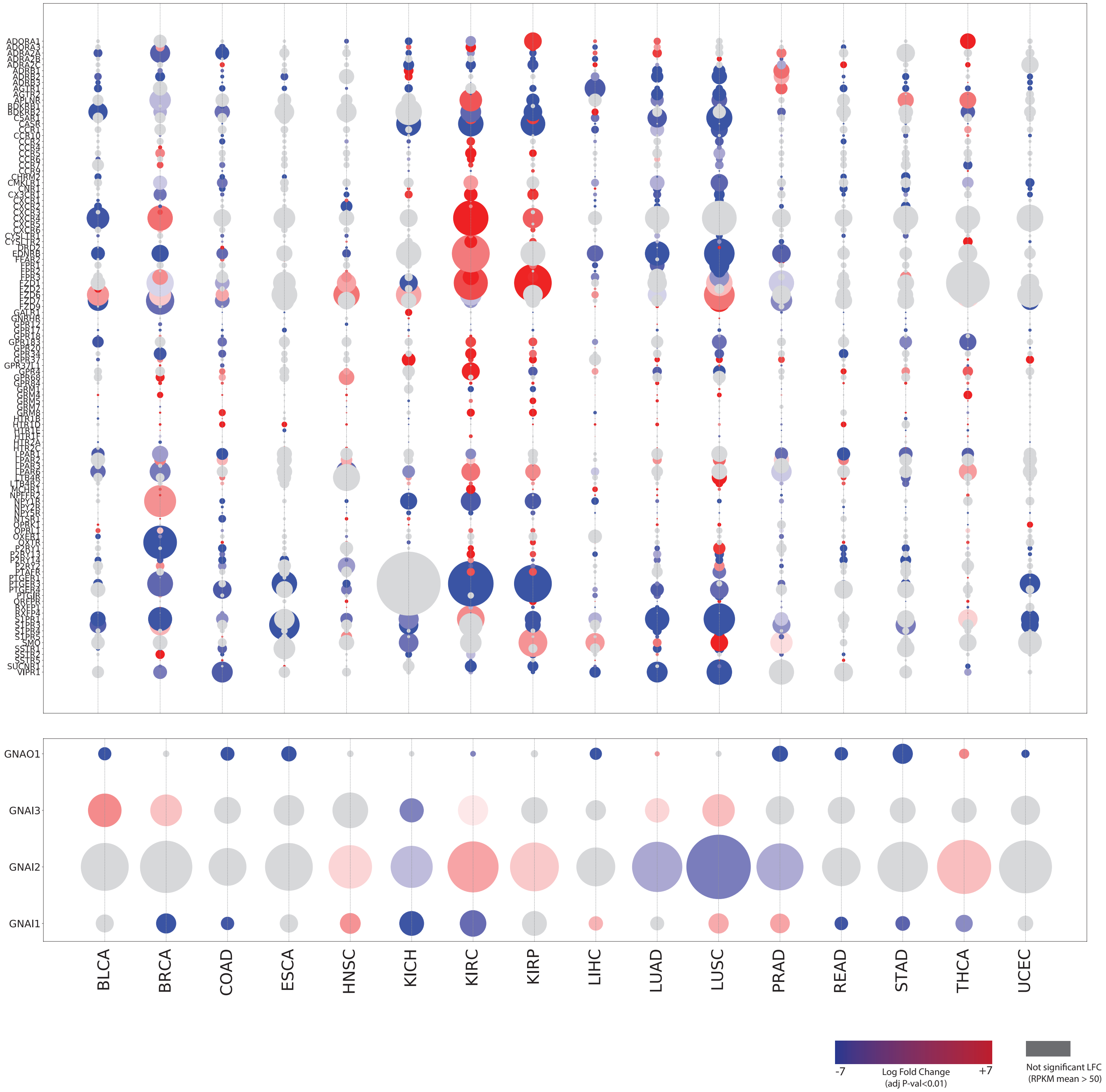

Figure S11

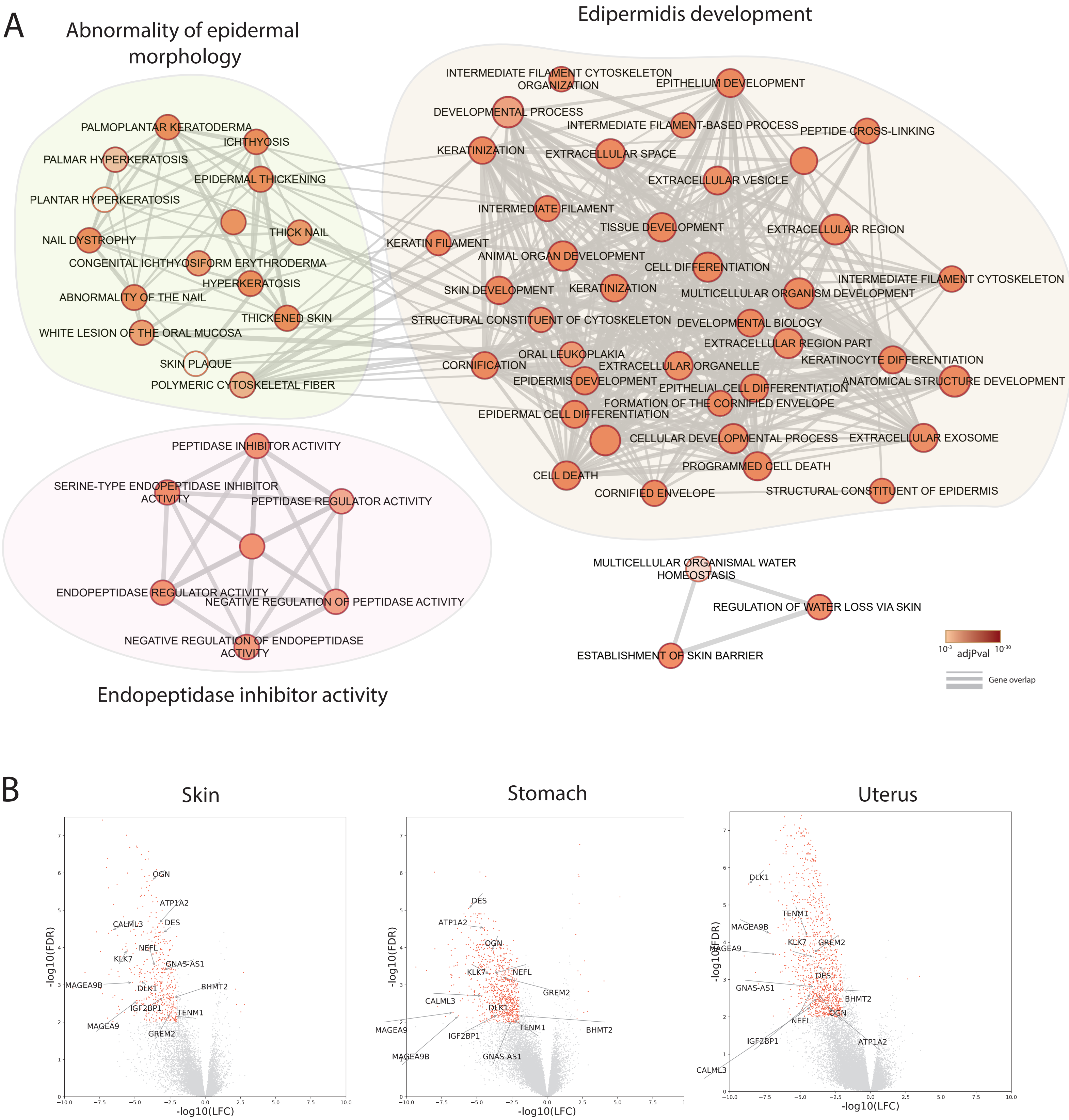
A
Abnormality of epidermal morphology
Edipermidis development
PALMOPLANTAR KERATODERMA
ICHTHYOSIS
PALMAR HYPERKERATOSIS
EPIDERMAL THICKENING
PLANTAR HYPERKERATOSIS
THICK NAIL
NAIL DYSTROPHY
CONGENITAL ICHTHYOSIFORM ERYTHRODERMA
HYPERKERATOSIS
ABNORMALITY OF THE NAIL
THICKENED SKIN
WHITE LESION OF THE ORAL MUCOSA
SKIN PLAQUE
POLYMERIC CYTOSKELETAL FIBER
INTERMEDIATE FILAMENT CYTOSKELETON ORGANIZATION
EPITHELIUM DEVELOPMENT
DEVELOPMENTAL PROCESS
INTERMEDIATE FILAMENT-BASED PROCESS
PEPTIDE CROSS-LINKING
KERATINIZATION
EXTRACELLULAR SPACE
EXTRACELLULAR VESICLE
INTERMEDIATE FILAMENT
TISSUE DEVELOPMENT
EXTRACELLULAR REGION
KERATIN FILAMENT
ANIMAL ORGAN DEVELOPMENT
CELL DIFFERENTIATION
INTERMEDIATE FILAMENT CYTOSKELETON
SKIN DEVELOPMENT
KERATINIZATION
MULTICELLULAR ORGANISM DEVELOPMENT
STRUCTURAL CONSTITUENT OF CYTOSKELETON
DEVELOPMENTAL BIOLOGY
EXTRACELLULAR REGION PART
ORAL LEUKOPLAKIA
KERATINOCYTE DIFFERENTIATION
CORNIFICATION
EXTRACELLULAR ORGANELLE
EPIDERMIS DEVELOPMENT
EPITHELIAL CELL DIFFERENTIATION
ANATOMICAL STRUCTURE DEVELOPMENT
FORMATION OF THE CORNIFIED ENVELOPE
EPIDERMAL CELL DIFFERENTIATION
CELLULAR DEVELOPMENTAL PROCESS
EXTRACELLULAR EXOSOME
PROGRAMMED CELL DEATH
CELL DEATH
CORNIFIED ENVELOPE
STRUCTURAL CONSTITUENT OF EPIDERMIS
PEPTIDASE INHIBITOR ACTIVITY
SERINE-TYPE ENDOPEPTIDASE INHIBITOR ACTIVITY
PEPTIDASE REGULATOR ACTIVITY
ENDOPEPTIDASE REGULATOR ACTIVITY
NEGATIVE REGULATION OF PEPTIDASE ACTIVITY
NEGATIVE REGULATION OF ENDOPEPTIDASE ACTIVITY
Endopeptidase inhibitor activity
MULTICELLULAR ORGANISMAL WATER HOMEOSTASIS
REGULATION OF WATER LOSS VIA SKIN
ESTABLISHMENT OF SKIN BARRIER
10-3 adjPval 10-30
Gene overlap
B
Skin
Stomach
Uterus
-log10(FDR)
-log10(LFC)
OGN
ATP1A2
CALML3
DES
NEFL
KLK7
GNAS-AS1
MAGEA9B
DLK1
BHMT2
IGF2BP1
TENM1
MAGEA9
GREM2

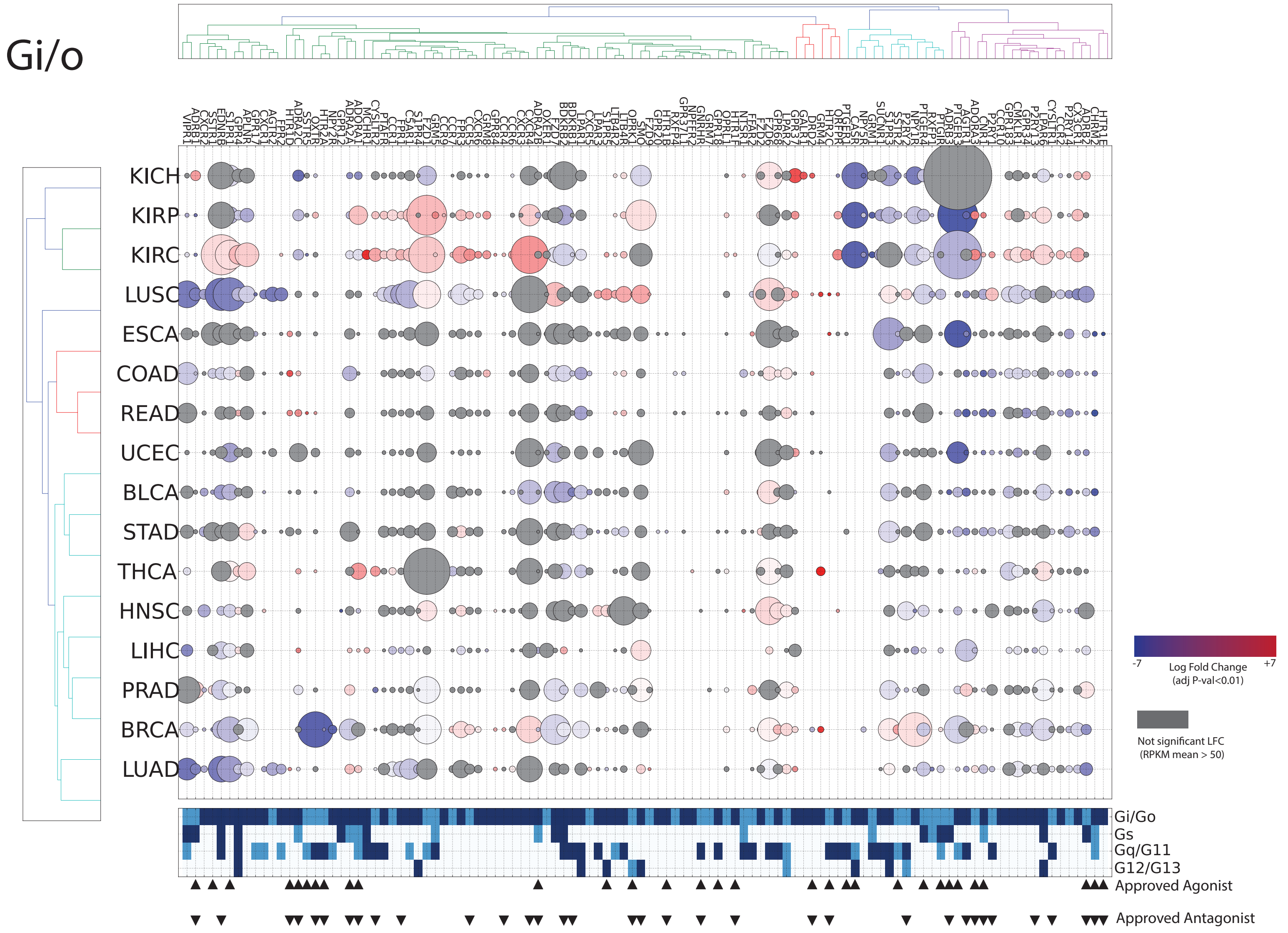
Gi/o
KICH
KIRP
KIRC
LUSC
ESCA
COAD
READ
UCEC
BLCA
STAD
THCA
HNSC
LIHC
PRAD
BRCA
LUAD
-7
Log Fold Change
(adj P-val<0.01)
+7
Not significant LFC
(RPKM mean > 50)
Gi/Go
Gs
Gq/G11
G12/G13
Approved Agonist
Approved Antagonist

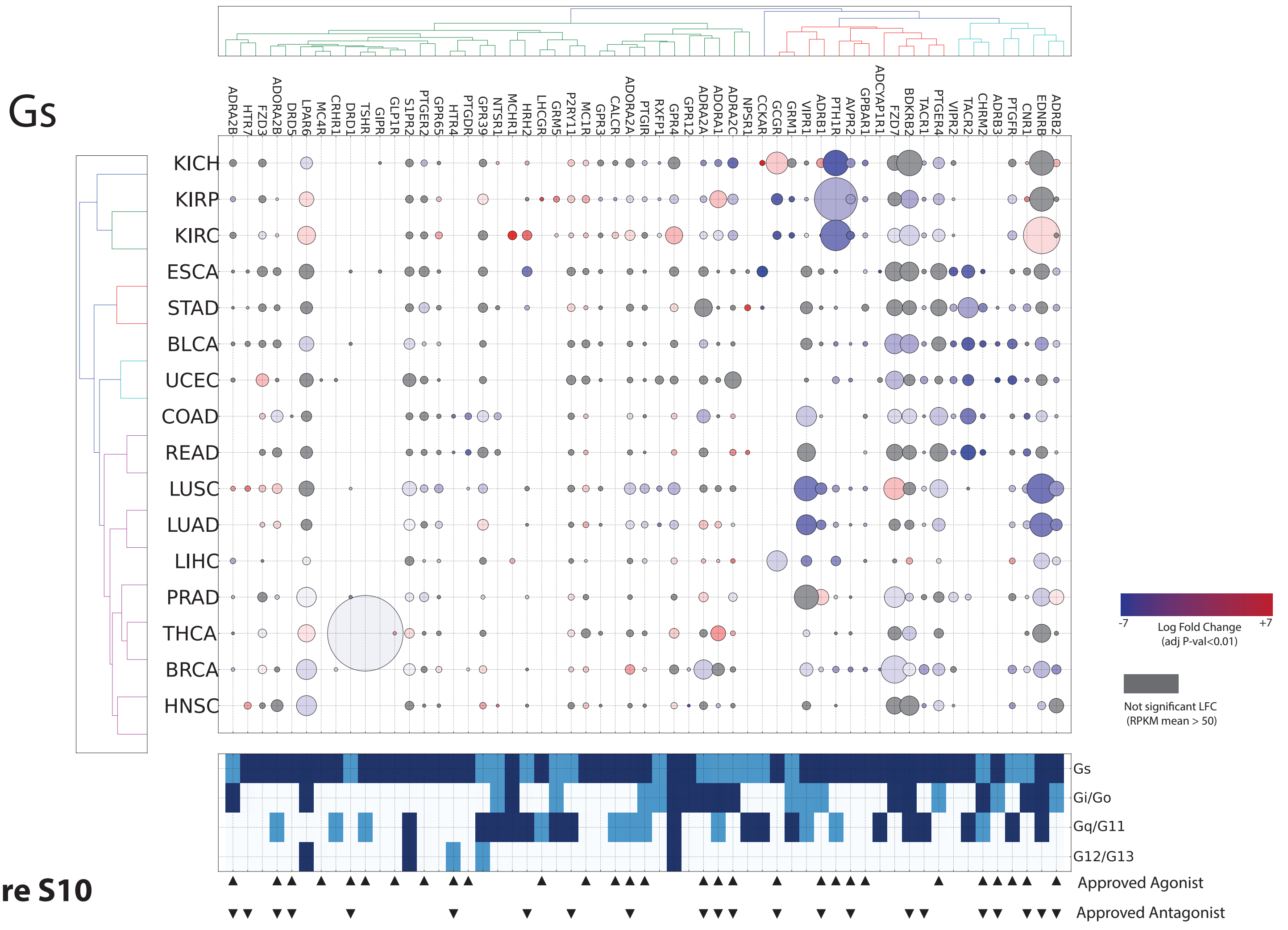
Gs
KICH
KIRP
KIRC
ESCA
STAD
BLCA
UCEC
COAD
READ
LUSC
LUAD
LIHC
PRAD
THCA
BRCA
HNSC
-7
Log Fold Change
(adj P-val<0.01)
+7
Not significant LFC
(RPKM mean > 50)
Gs
Gi/Go
Gq/G11
G12/G13
Approved Agonist
Approved Antagonist

Figure S10

A

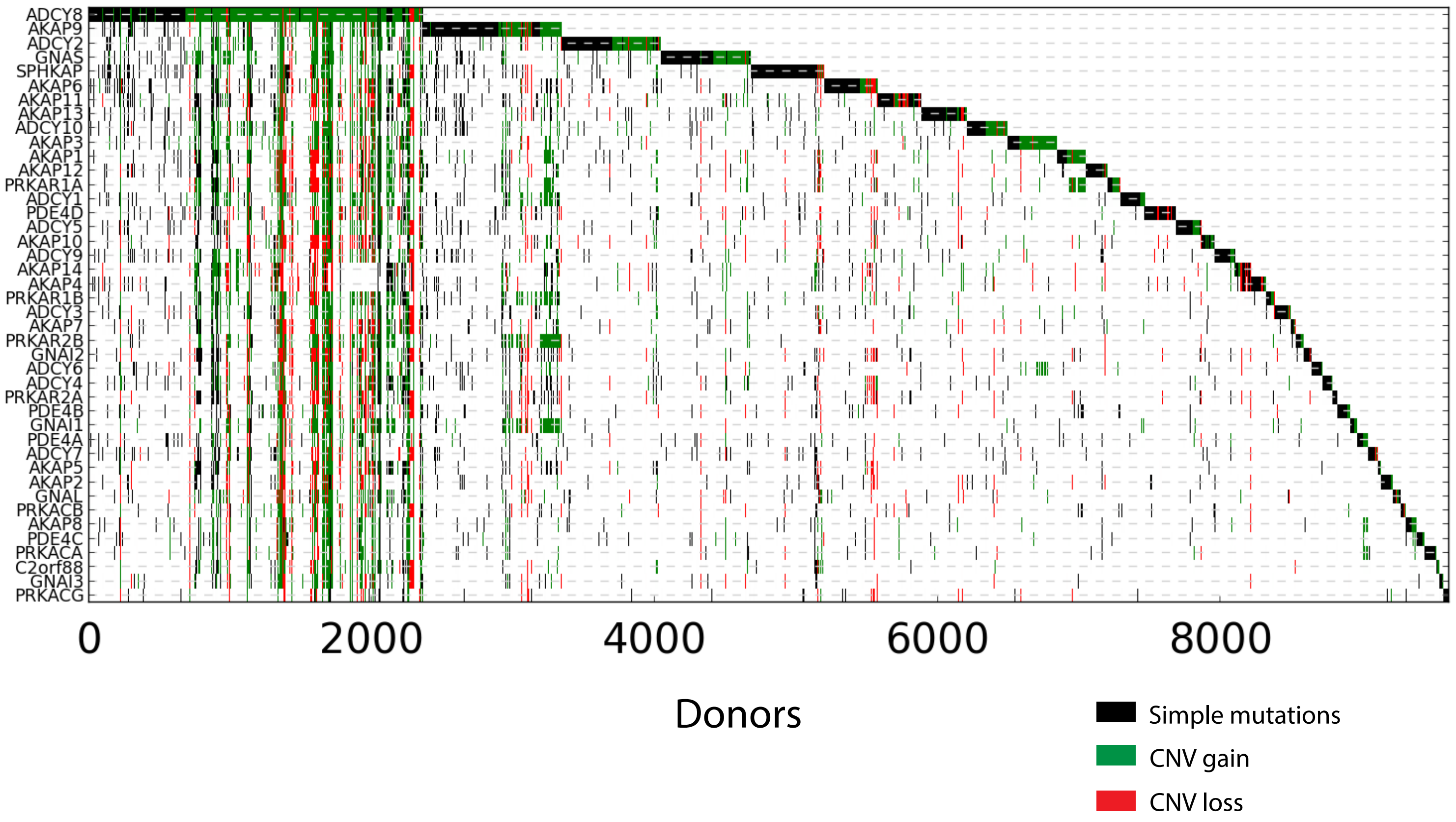

ADCY8
AKAP9
ADCY2
GNAS
SPHKAP
AKAP6
AKAP11
AKAP13
ADCY10
AKAP3
AKAP1
AKAP12
PRKAR1A
ADCY1
PDE4D
ADCY5
AKAP10
ADCY9
AKAP14
AKAP4
PRKAR1B
ADCY3
AKAP7
PRKAR2B
GNAI2
ADCY6
ADCY4
PRKAR2A
PDE4B
GNAI1
PDE4A
ADCY7
AKAP5
AKAP2
GNAL
PRKACB
AKAP8
PDE4C
PRKACA
C2orf88
GNAI3
PRKACG
0
2000
4000
6000
8000
Donors
Simple mutations
CNV gain
CNV loss

B

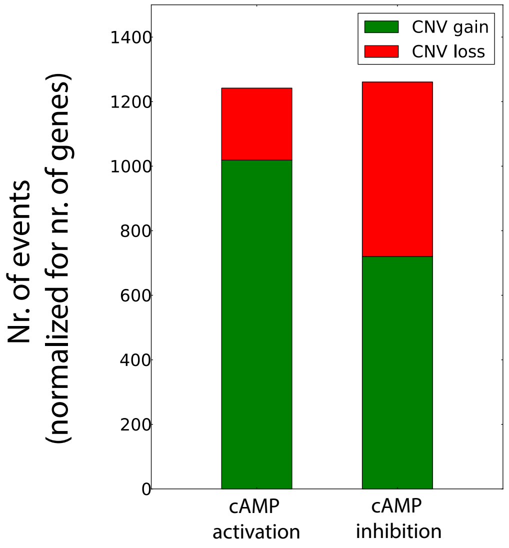

Nr. of events
(normalized for nr. of genes)
0
200
400
600
800
1000
1200
1400
CNV gain
CNV loss
cAMP
activation
cAMP
inhibition
