# Supplementary figures and images for: Rare, functional, somatic variants in gene families linked to cancer genes: GPCR signaling as a paradigm
Source: Oncogene. 2019 Jul 23;38(38):6491–506. doi: 10.1038/s41388-019-0895-2 (PMC6756116; doi:10.1038/s41388-019-0895-2)

A

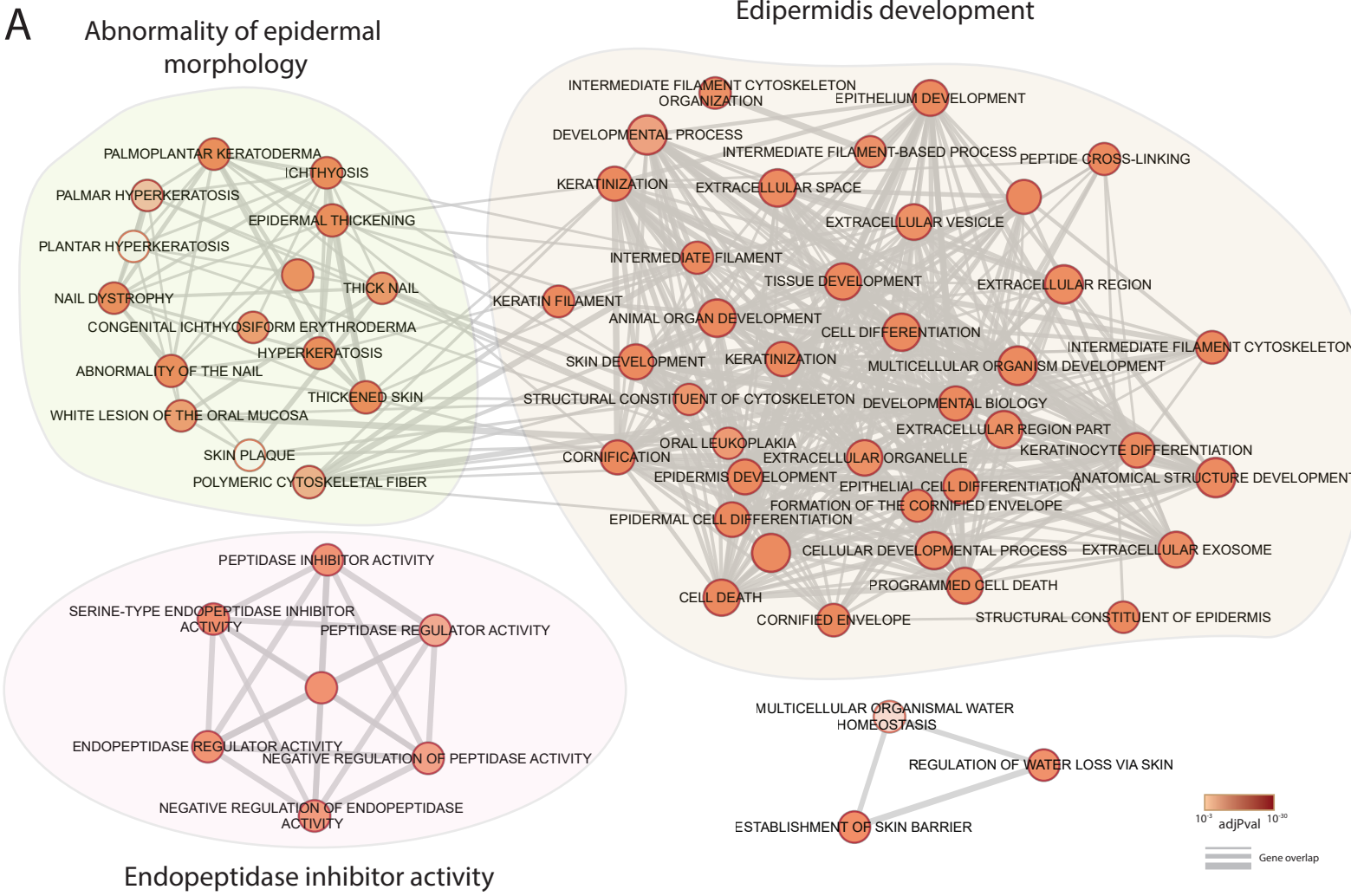

B

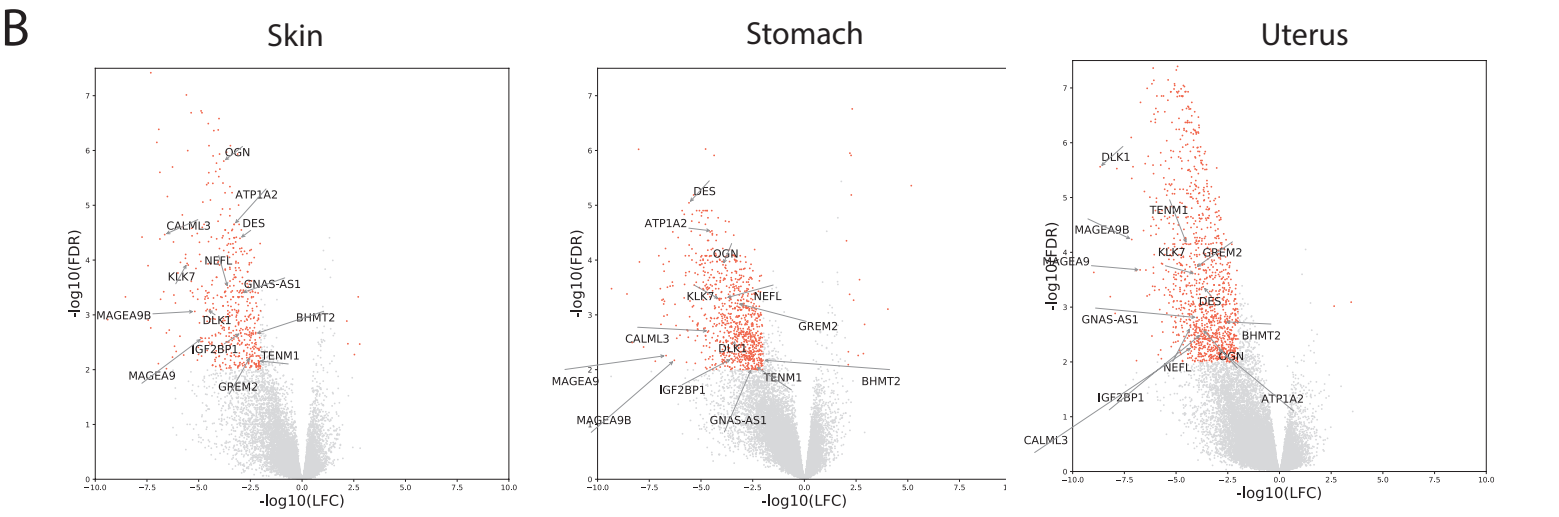

Figure S12

Supplement: Supplementary file 4 — Figure_S12 [file 41388_2019_895_MOESM4_ESM.pdf]

**A**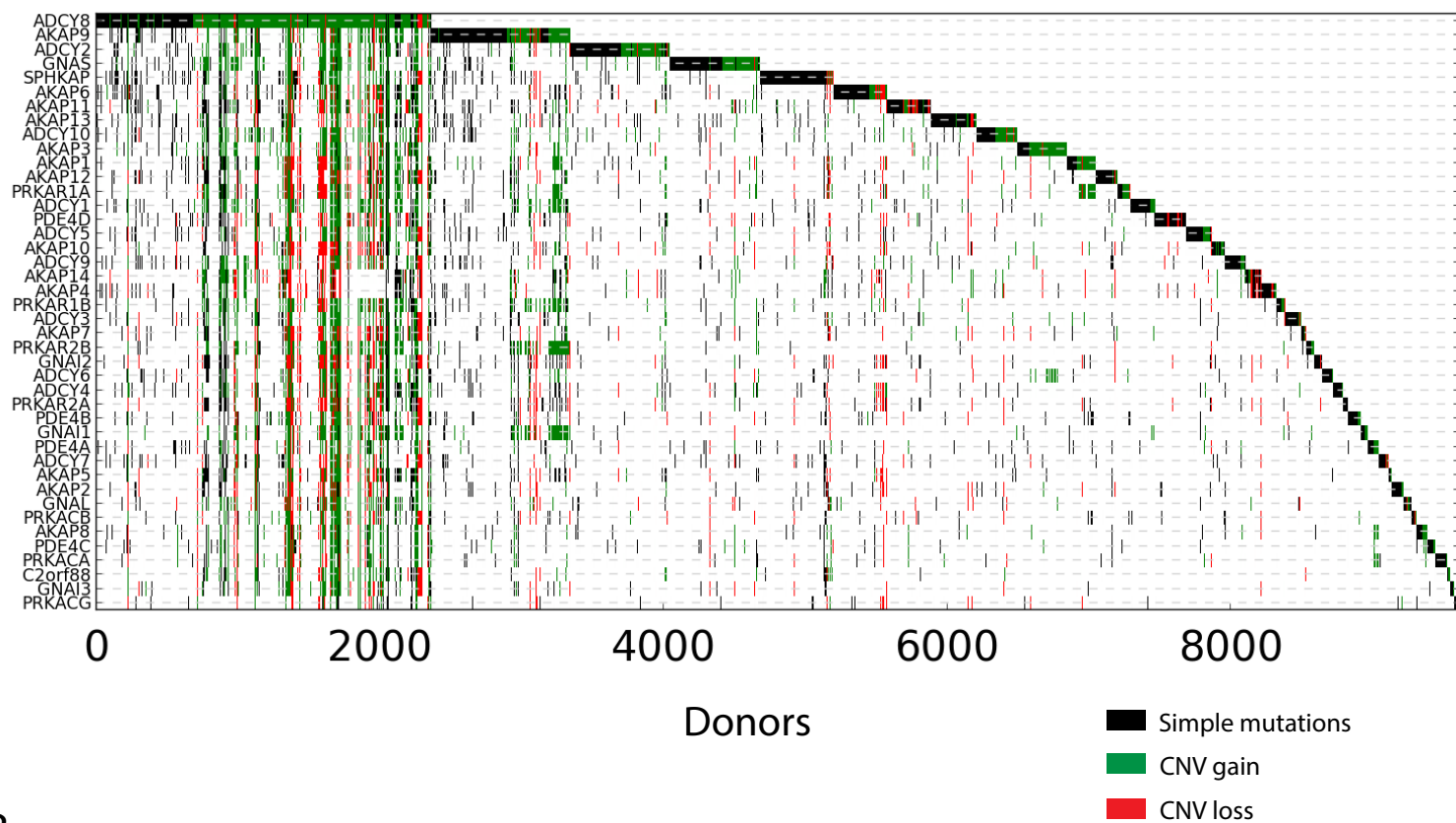**B**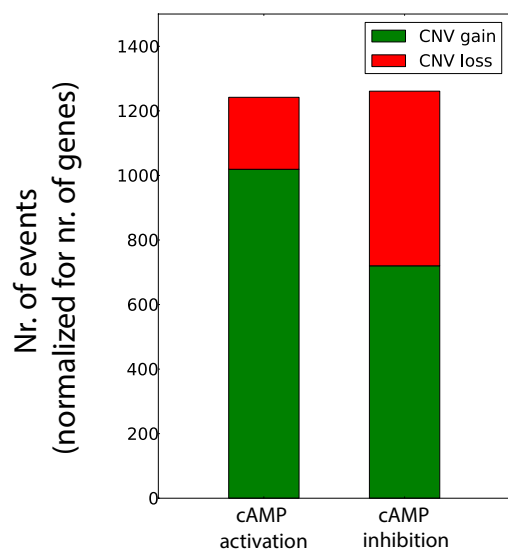**Figure S14**

Supplement: Supplementary file 6 — Figure_S14 [file 41388_2019_895_MOESM6_ESM.pdf]
