# Supplementary material for: Rare, functional, somatic variants in gene families linked to cancer genes: GPCR signaling as a paradigm
Source: Oncogene. 2019 Jul 23;38(38):6491–506. doi: 10.1038/s41388-019-0895-2 (PMC6756116; doi:10.1038/s41388-019-0895-2)
Supplement: Supplementary file 5 — Figure_S13 [file 41388_2019_895_MOESM5_ESM.pdf]

Gi/o

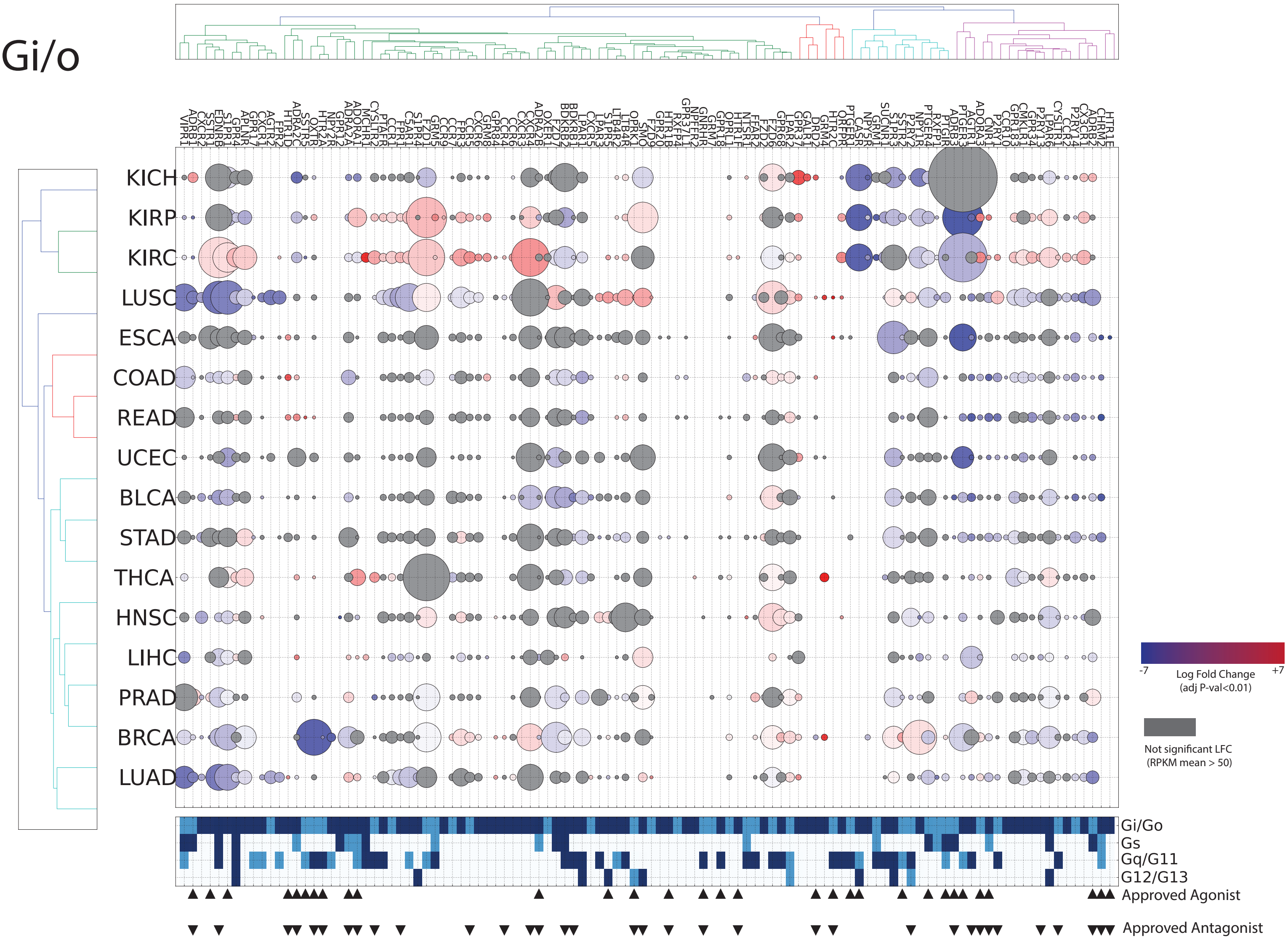

Gs

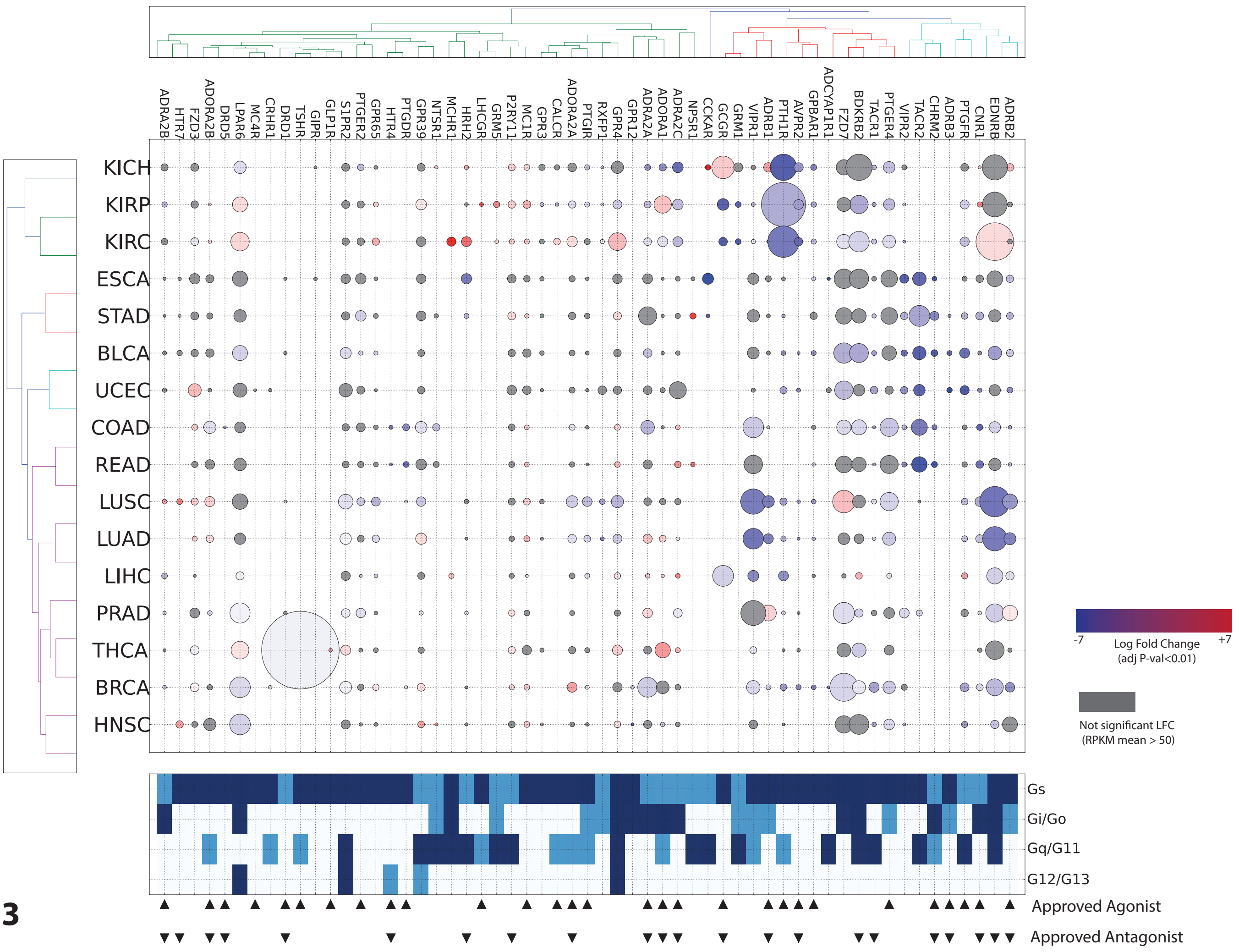

Figure S13
